# Anti-tumor effects of dual PI3K-HDAC inhibitor CUDC-907 on activation of ROS-IRE1α-JNK-mediated cytotoxic autophagy in esophageal cancer

**DOI:** 10.1186/s13578-022-00855-x

**Published:** 2022-08-21

**Authors:** Zheng Jian, Yichao Han, Wentian Zhang, Chengqiang Li, Wei Guo, Xijia Feng, Bin Li, Hecheng Li

**Affiliations:** 1 Department of Thoracic Surgery, Ruijin Hospital, Shanghai Jiao Tong University School of Medicine, Shanghai, China; 2 Department of Thoracic Surgery, Shanghai Pulmonary Hospital, Tong Ji University School of Medicine, Shanghai, China; 3 Shanghai Institute of Immunology, Department of Immunology and Microbiology, Shanghai Jiao Tong University School of Medicine, Shanghai, China

**Keywords:** PI3K-Akt, HDAC, CUDC-907, Autophagy, Esophageal cancer, CDX

## Abstract

**Background:**

PI3K-Akt pathway activation and the expression of histone deacetylases (HDACs) are highly increased in esophageal cancer, suggesting that inhibition of such targets may be a viable therapeutic strategy. Herein, we aimed to evaluate the anti-tumor effect of CUDC-907, a dual PI3K-HDAC inhibitor, in esophageal squamous cell carcinoma (ESCC).

**Methods:**

The anti-tumor effects of CUDC-907 in ESCC were evaluated using cell counting kit-8, flow cytometry, and western blot. mRNA-sequencing was used to explore the mechanism underlying CUDC-907 anti-tumor effects. The relations of reactive oxygen species (ROS), lipocalin 2 (LCN2), and CUDC-907 were determined by flow cytometry, rescue experiments, and western blot. The activation of the IRE1α-JNK-CHOP signal cascade was confirmed by western blot. The in vivo inhibitory effects of CUDC-907 were examined by a subcutaneous xenograft model in nude mice.

**Results:**

CUDC-907 displayed effective inhibition in the proliferation, migration, and invasion of ESCC cells. Through an mRNA-sequencing and functional enrichment analysis, autophagy was found to be associated with cancer cells death. CUDC-907 not only inhibited the PI3K-Akt-mTOR pathways to result in autophagy, but also induced ROS accumulation to activate IRE1α-JNK-CHOP-mediated cytotoxic autophagy by downregulating LCN2 expression. Consistently, the in vivo anti-tumor effects of CUDC-907 accompanied by the downregulated expression of p-mTOR and LCN2 and upregulated expression of p-IRE1α and LC3B-II were evaluated in a xenograft mouse model.

**Conclusion:**

Our findings suggested the clinical development and administration of CUDC-907 might act as a novel treatment strategy for ESCC. A more in-depth understanding of the anti-tumor effect of CUDC-907 in ESCC will benefit the clinically targeted treatment of ESCC.

**Supplementary Information:**

The online version contains supplementary material available at 10.1186/s13578-022-00855-x.

## Background

Esophageal cancer is reportedly one of the most common malignant tumors of the digestive tract, severely threatening human health [1]. According to recent studies, esophageal cancer is the sixth most common cause of cancer‐related death worldwide, and esophageal squamous cell carcinoma (ESCC) is the predominant pathological type [1–3]. Although advances in the early diagnosis, radical operations, and neoadjuvant therapies have partially improved the prognosis of patients with esophageal cancer, the 5‐year overall survival rate remains only 20–40% [4]. Hence, molecular-level studies of esophageal cancer and its pathogenesis are needed to develop effective intervention strategies.

Carcinogenesis of esophageal epithelial cells is a multistage process associated with many critical genetic or signaling pathway-related alterations [2, 5]. Among them, the abnormal upregulation of the PI3K/Akt/mTOR pathway regulates cellular survival, proliferation, migration, invasion, autophagy, and metabolism homeostasis [6–8]. Investigation of inhibitors targeting mTOR demonstrated that the development of drugs related to the PI3K/Akt/mTOR pathway is promising for treating esophageal cancer [8]. Additionally, histone deacetylases (HDACs) are commonly overexpressed in esophageal cancer [9, 10]. As HDAC inhibitors are widely implemented for cancer therapy, their effects on the induction of apoptosis, differentiation, autophagy, and cell cycle arrest have been reported in different tumor types [11–13]. Indeed, the emergence of cancer therapy targeting this signaling cascade has brought hope for patients with esophageal cancer. Nevertheless, most patients develop drug resistance or do not respond to single-targeted agents [14, 15].

To address these problems, CUDC-907, an innovative artificial small-molecule inhibitor, has been formulated by combining the PI3K-Akt inhibitory functionality with the pharmacophore of HDAC inhibitors [14]. Previous studies reported the potential efficiency of CUDC-907 in the treatment of lymphoma and other solid tumors [15–17], while its impacts on esophageal cancer remain unclear. Our research comprehensively investigates the anti-tumor effect of CUDC-907 in vitro and in vivo, suggesting the efficacious inhibition of CUDC-907 on the proliferation, migration, and invasion of esophageal cancer cells and the induction autophagy via mTOR inhibition and LCN2 downregulation.

## Materials and methods

### Cell cultures and tissue samples

Human ESCC cell lines (TE-1, KYSE-150, KYSE-450, KYSE-510, KYSE-30, and KYSE-180) and normal human esophageal epithelial cells (Het-1A) were purchased from the National Collection of Authenticated Cell Culture in the Chinese Academy of Sciences (Shanghai, China). All purchased cells were identified and characterized as previously described [14].

Cells were cultured in Roswell Park Memorial Institute (RPMI) 1640 medium (2.0 g/L of D-glucose and 0.3 g/L of L-glutamine) containing 10% fetal bovine serum,1% penicillin–streptomycin, and 1% non-essential amino acids in 5% CO_2_ incubators at 37 °C.

Human esophageal tissue samples were collected from six patients with operable ESCC (Additional file 1: Fig. S1a) based on a clinical protocol approved by the Ethics Committee of Ruijin Hospital affiliated with Shanghai Jiao Tong University, School of Medicine (KY201996). All patients provided informed consent. The obtained esophageal tissue samples were stored at − 80 °C.

### Reagents

CUDC-907, rapamycin (mTOR inhibitor), and trichostatin A (HDAC inhibitor) were purchased from Selleck Chemicals (Houston, TX, USA). Pictilisib (PI3K-Akt inhibitor), carboplatin, paclitaxel, 3-methyladenine (3-MA, autophagy inhibitor), chloroquine (CQ, autophagy inhibitor), MHY1485 (mTOR activator), and SP600125 (JNK inhibitor) were purchased from MedChemExpress (Monmouth Junction, NJ, USA). N-acetyl-L-cysteine (NAC) was purchased from Sigma-Aldrich (St. Louis, MO, USA). CUDC-907 powder was dissolved at 10 M in pure dimethyl sulfoxide (DMSO) and stored at − 20 °C. Control cells were treated with DMSO as a vehicle.

### Cell proliferation and viability assay

Cell proliferation and viability assay were implemented by cell counting kit-8 (CCK-8; Beyotime, Shanghai, China). Cells were seeded at a density of 2000 cells/100 µL in quintuplicate in 96-well opaque plates. The next day, cells were supplemented with 100 µL of fresh medium containing different concentrations of CUDC-907, pictilisib, trichostatin A, carboplatin, or their combinations for 72 h. For autophagic flux experiments, ESCC cells were exposed to 3-MA (5 mM) or CQ (20 µM) for 6 h and then treated with CUDC-907 (30 nM). Rapamycin (5 nM) and MHY1485 (10 µM) were applied to modulate mTOR activation for 48 h. SP600125 (10 µM) was added to suppress the JNK signal cascade. NAC (5 mM) was added for 6 h before subsequent treatments. siRNA methods were used to knock down the expression of key targets (ULK1, LCN2, and IRE1α). After being transfected with siRNA, cells were collected and exposed to CUDC-907 (Selleck, 30 nM) for 48 h, or priorly treated with NAC (5 mM) for siLCN2. CCK-8 buffer was added at 1:10 to the RPMI 1640 medium for the experiment. After incubation for 3 h, the optical density values in each duplicate well were measured by a spectrophotometer (Molecular Devices, Sunnyvale, CA, USA) at 450 nm.

### Lactate dehydrogenase assay

Cells were seeded at 2000 cells/100 µL in quintuplicate in 96-well opaque plates. LDH release assay kit (Beyotime, Shanghai, China) was used to evaluate the lactate dehydrogenase (LDH) activity in the cell culture medium following the manufacturer’s instructions. Briefly, 60 μL working solution was added into each duplicate well after the treatment with the drugs or siRNA. Then, the plates were incubated in dark condition at room temperature for 30 min. The LDH activity was confirmed by measuring the optical density at 490 nm using a spectrophotometer.

### Cell cycle and apoptosis assay

Cells were seeded at a density of 1 × 10^5^ in a 6-well dish, treated with vehicle or CUDC-907 (10 and 30 nM) for 24 h, and collected by 0.25% trypsin–EDTA. To determine the cell cycle distribution, a Cell Cycle and Apoptosis Analysis Kit (Beyotime, Shanghai, China) was applied according to the manufacturer’s instructions. Specifically, cells were detached and centrifuged at 1000×*g* for 10 min, and then fixed in 500 μL pre-cooled 70% ethanol for at least 6 h. Next, 10 × propidium iodide (PI) solution was used to stain cells at room temperature for 20–30 min. A flow cytometry (BD Biosciences, Franklin Lakes, NJ, USA) and the ModiFit LT v2.0 program were used for flow cytometric analysis. To identify apoptosis rate, an Annexin V/PI Apoptosis Kit (BD Biosciences Franklin Lakes, NJ) was employed to distinguish the different statuses of apoptotic cells. In general, Annexin V^+^/PI^−^ represents the early stage of apoptosis, whereas Annexin V^+^/PI^+^ indicates the late stage of apoptosis. Cells were incubated at room temperature for 20 min and analyzed via flow cytometry.

### Cellular migration and invasion assay

A wound-healing experiment was used to evaluate the inhibitory effect of CUDC-907 on the migratory capacity of ESCC cells. Once ESCC cells cultured in 6-well dishes reached 85–95% confluency, they were then scratched using a 200-μL pipette tip, and cells were then cultured in serum-free medium with vehicle or CUDC-907 (10 and 30 nM) for 24 h. Photographs were taken at 0 h and 24 h after scratching. The breadth of blank areas was measured by Photoshop software (Adobe Photoshop CC 2018). The percentage of the area filled with cells was calculated using the following formula: cellular migration rate (%) = ((breadth 0 h − breadth 24 h)/breadth 0 h) × 100.

The cellular invasion ability was determined using a Transwell chamber (8-μm pore size, Corning, Inc., Corning, NY, USA) following the manufacturer’s instructions. Cells were treated with vehicle or CUDC-907 (10 and 30 nM) for 24 h and then seeded in the upper Transwell chamber, which was covered with 20–50 μL Matrigel, at a density of 50,000 cells/mL in serum-free medium with a total volume of 300 μL. In the bottom chamber, 750 μL of RPMI 1640 supplemented with 10% FBS were added as a chemoattractant. Cells were incubated at 37 °C for 48 h, and invading cells were fixed and stained with crystal violet staining. Cells were then photographed and analyzed using ImageJ software (Version 1.8.0, National Institutes of Health, Bethesda, MD, USA).

### Immunohistochemical (IHC) and immunofluorescence staining

Tissues were fixed with 4% formalin overnight and then dehydrated and paraffin-embedded. Section separation was followed by continuous rehydration. Antigen repair was performed with 1× citrate buffer at 120 °C, followed by sealing of the sections and overnight incubation at 4 °C for antibody immunostaining. Afterward, 3,3′-diaminobenzidine (G1211, Servicebio, Wuhan, China), with a GTVision TM III detection system/Mo&Rb (K5007, Dako, Glostrup, Denmark), were applied to determine the immunoreactivity of the primary antibody. Antibodies against the following proteins were used in this study: HDAC2 (1:500, Cell Signaling Technology (CST), #5113), p-Akt (1:500, CST, #4060), p-mTOR (1:500, CST, #4060), p-IRE1 (1:500, Novus, NB100-2323), LCN2 (1:200, Abcam, ab125075). Three random fields for each sample were captured at 10× and 40× magnification using a CICXSP-C204 microscope (NIKON).

Immunofluorescence staining was performed following standard procedures. Adherent cells were fixed onto glasses using 1% paraformaldehyde. After blocking and permeabilizing, cells were incubated with anti-γ-H2AX (1:500, Abcam, ab81299) followed by a fluorophore-conjugated secondary antibody for 1 h in dark humidified conditions. After washing, cells were counterstained using a SlowFade Gold anti-fade reagent with DAPI (4′,6-diamidino-2-phenylindole) (Invitrogen, Carlsbad, CA) and imaged using a fluorescence microscope.

### Western blot analysis

Total protein was extracted from ESCC cells after different kinds of treatment by utilizing RIPA lysis buffer, containing inhibitors of protease and phosphatase. Cell lysates were separated using SDS-PAGE and transferred to NC or PVDF membranes. Primary antibodies against the proteins mentioned in our research are described in additional materials (Additional file 1: Additional Material S1. Rabbit IgG (1:3000 #7074) and mouse IgG (1:3000 #7076) were purchased from CST for the application of secondary antibody.

### ROS generation and GFP-LC3 puncta assay

The generation of intracellular ROS was determined with a cell-permeable probe, dichloro-dihydro-fluorescein diacetate (DCFH-DA) (Beyotime, Shanghai, China). Following treatment with CUDC-907 or NAC + CUDC-907, cells were incubated with DCFH-DA (1:1000) in the dark at room temperature for at least 20 min and analyzed via flow cytometry. The same procedures were used in the siLCN2 and LCN2-overexpressing ESCC cells.

ESCC cells with a stable expression of green fluorescent protein (GFP)-microtubule-associated protein 1A/1B-light chain 3 (LC3) were seeded in 6-well dishes. After exposure to vehicle or CUDC-907 (10 nM and 30 nM), cells were washed with PBS at least three times and fixed with 1% paraformaldehyde. After that, cells were rinsed with PBS at least three times to remove paraformaldehyde and then stained with DAPI in the dark. Three random fields at 10× and 40× magnification were captured for each sample using a fluorescence microscope.

### ChIP-qPCR assay and intracellular iron detection

ChIP-qPCR assays were performed using Chromatin Extraction Kit (ab117152, Abcam) and ChIP Kit–One Step (ab117138, Abcam) as described in the supplier’s protocol. Antibodies used in this assay were purchased from Abcam, including H3 (acetyl K9) ChIP grade (ab177178), H3 (acetyl K27) ChIP grade (ab32129). ChIP-qPCR primers were showed in Table 1. Quantitative PCR conditions were 40 cycles at 94 °C for 40 s, 60 °C for 1 min, and 72 °C for 40 s.

**Table 1:** ChIP qPCR primers

Gene	Primer sequence (5′–3′)
Acetyl(K9)-LCN2	Sense-1: CAGCCATCTCGGTGACAGTT
	Antisense-1: GAGACCATCCCCCTGACTCT
	Sense-2: GCCATCTCGGTGACAGTTCC
	Antisense-2: ACTGAGACCATCCCCCTGAC
Acetyl(K27)-LCN2	Sense-1: GGTGACAGTTCCAGAACCCC
	Antisense-1: CAGTGCAAGGATCTGGCCTT
	Sense-2: CCTCAGCACTCAACCCATGT
	Antisense-2: CCCTCTCCTTGCCCCATTTG

To evaluate the level of intracellular reductive iron ions, we used FerroOrange (F374, Dojindo) kit. Generally speaking, KYSE-450 and KYSE-510 cells were seeded at a density of 5000 cells/100 µL in quintuplicate in 96-well opaque plates. The next day, the cells were supplemented with 100 µL of fresh medium containing different concentrations of CUDC-907 (0, 10, 30 nM). After 6 h cultivation in 5% CO2 incubators at 37 °C, use fresh FBS-free medium to wash 3 times. After that, add 1 μmol/L FerroOrange working solution into the fresh FBS-free medium and co-culture with the cells treated with different concentrations of CUDC-907 for 30 min. The values of optical density in each duplicate well were measured by a spectrophotometer (Molecular Devices, Sunnyvale, CA, USA).

### RNA-sequencing (RNA-seq) and database analysis

Total RNA was extracted and quantified according to the manufacturer’s instructions. RNA-seq libraries of these RNA samples were constructed based on the technical protocols. The final libraries were amplified with BioAnalyzer 2100 system (Agilent Technologies, Inc., USA). Next, 10 pM libraries were denatured as single-stranded DNA molecules, captured on Illumina flow cells, amplified in situ as clusters, and finally sequenced for 150 cycles on Illumina NovaSeq 6000 Sequencer according to the manufacturer’s instructions. Differentially expressed genes were selected according to the criteria of |log_2_FC| ≥ 2, adjusted P-value ≤ 0.01. The data relating to the expression of HDAC1 to 9 in normal tissues compared to tumor tissues were downloaded from The Cancer Genome Atlas (TCGA) database. The database analysis was performed using R (version 4.1.2).

### Animal studies

All experimental protocols involving animals were approved by the Animal Care & Welfare Committee of Shanghai Ruijin Hospital affiliated Shanghai Jiao Tong University School of Medicine. To verify the effect of CUDC-907 on esophageal cancer, a xenograft model was established. KYSE-450 cells resuspended with PBS were injected subcutaneously into the axilla of C57BL/6 nude male mice aged from six to eight weeks. After the subcutaneous tumors were formed (day 10), mice were randomly divided into two groups: vehicle group (n = 6) and 200 mg/kg CUDC-907 group (n = 6). Mice in the vehicle group (control group) were fed with 30% Captisol, whereas treatment mice (200 mg/kg CUDC-907 group) were fed with 200 mg/kg CUDC-907 dissolved in 30% Captisol. The dose regimen lasted 5 days, followed by 2 days without medication, which was orally gavaged. Size of tumors and mice weight were recorded every third day. On day 31, all mice were euthanized, primary tumor tissues were collected for the western blot analysis, and some other primary tumors were fixed using 1% paraformaldehyde, then dehydrated and paraffin-embedded. Section separation was followed by continuous rehydration for immunohistochemical staining. Histochemistry score (H-score) calculation was performed by two experienced pathologists according to their clinical protocols. Briefly, the grading of positive intensity area was classified as follows: negative—0; weak intensity area—1; moderate intensity area—2; strong intensity area—3. H-Score = (percentage of weak intensity area × 1) + (percentage of moderate intensity area × 2) + (percentage of strong intensity area × 3).

### Statistical analysis

All in vitro analytical experiments were repeated at least three times. Error bars indicated the standard deviation (SD). Statistical significance was confirmed by Student’s t-test. One-way ANOVA was applied to judge significant differences among multiple groups. All statistical analyses and IC_50_ value calculations were conducted via the GraphPad Prism version 8.3.1. Thresholds of P-value significance are as follows: *P < 0.05; **P < 0.01; ***P < 0.001.

## Results

### Upregulated PI3K-Akt pathways and HDACs in patients with ESCC and cell lines

**Fig. 1:**
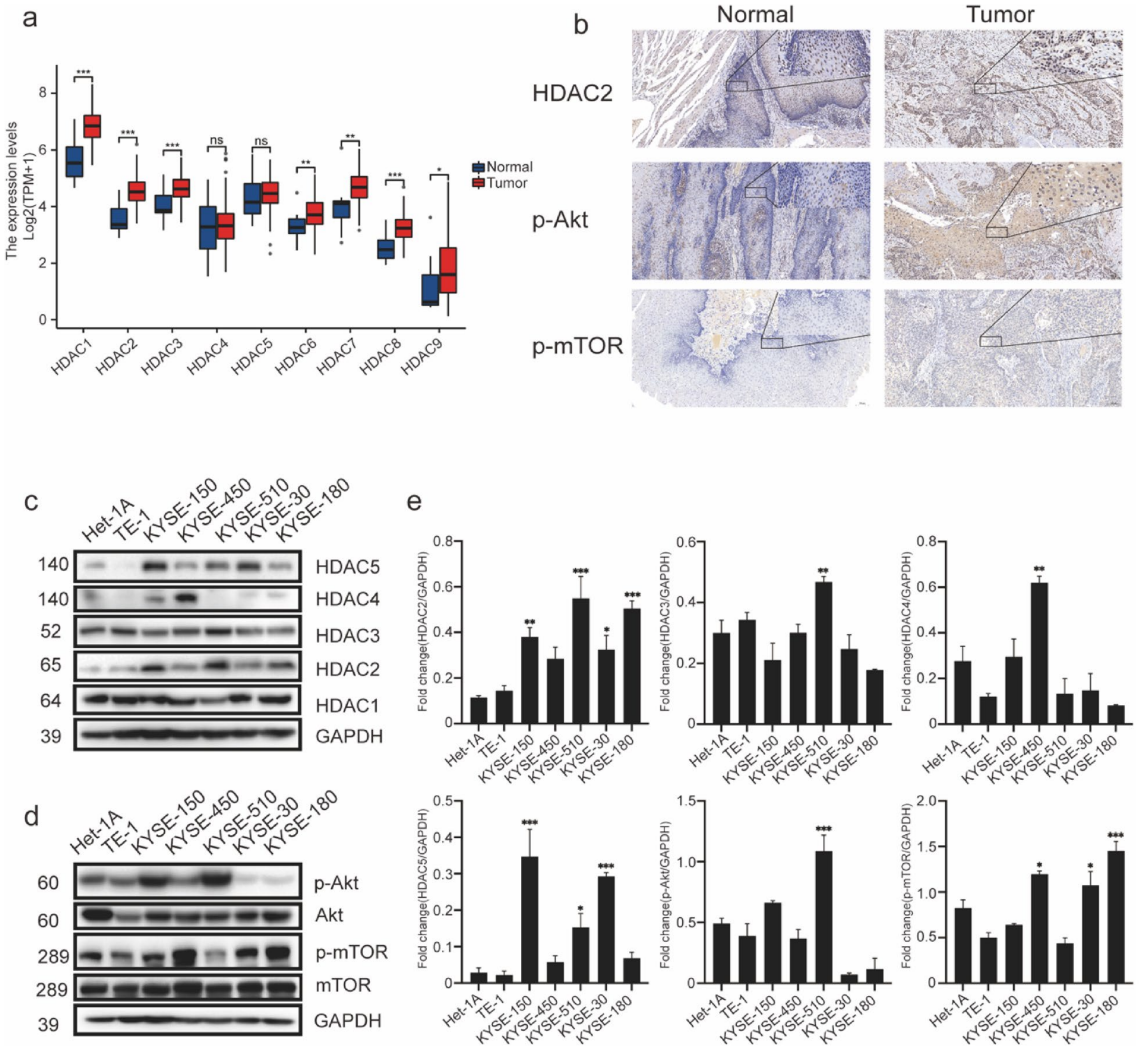
PI3K-Akt pathways and HDACs are upregulated in ESCC patients and cell lines. The expressions of HDACs, p-Akt/Akt, and p-mTOR/mTOR were tested in ESCC patients or cell lines. **a** The different expressions of mRNA levels related to HDACs in normal esophageal tissues and esophageal cancers from TCGA databases were compared in pairs. **b** Immunohistochemical (IHC) staining methods detected the protein levels of HDAC2, phosphorylation of Akt, and mTOR in normal esophageal tissues and esophageal cancers. Scale bar: 20 μm and 100 μm. **c**, **d** Western blot analysis of HDACs, p-Akt/Akt and p-mTOR/mTOR in different ESCC cell lines. **e** Statistical analysis of upregulated proteins of ESCC cells vs. normal esophageal epithelial (Het-1A) cells by western blot. Error bars are ± SD. *P < 0.05; **P < 0.01; ***P < 0.001

The PI3K-Akt pathway has been shown to promote the survival and proliferation of various types of cancers [7], and HDACs, which are important for transcriptional gene activity, are involved in multiple stages of carcinogenesis [18, 19], particularly class I HDACs [20]. Therefore, we first explored the expression of HDAC genes in esophageal cancer using TCGA data. The results showed that HDAC1, 2, 3, 6, 7, 8, and 9 were significantly upregulated in tumor tissues compared to that in normal tissues (Fig. 1a). Using tissues from patients (Additional file 1: Fig. S1a, b) with operable ESCC, the expression of HDAC2 was verified by IHC (Fig. 1b). Afterward, we evaluated the expression of phosphorylation of Akt (Ser473) (p-Akt) and one of its key downstream molecules, p-mTOR (Ser2448) by IHC. We observed that these two molecules were significantly upregulated in tumors (Fig. 1b). To verify the overexpression of HDACs, p-Akt/Akt, and p-mTOR/mTOR in ESCC cell lines, we took the normal human esophageal epithelial cell line, Het-1A, as a control. Western blot analysis was performed, and using Image J, we showed that HDAC2, HDAC3, HDAC4, HDAC5, p-Akt, and p-mTOR were upregulated in different types of ESCC cells (Fig. 1c–e).

### Apoptosis and G2/M arrest and inhibition of cell proliferation by CUDC-907

**Fig. 2:**
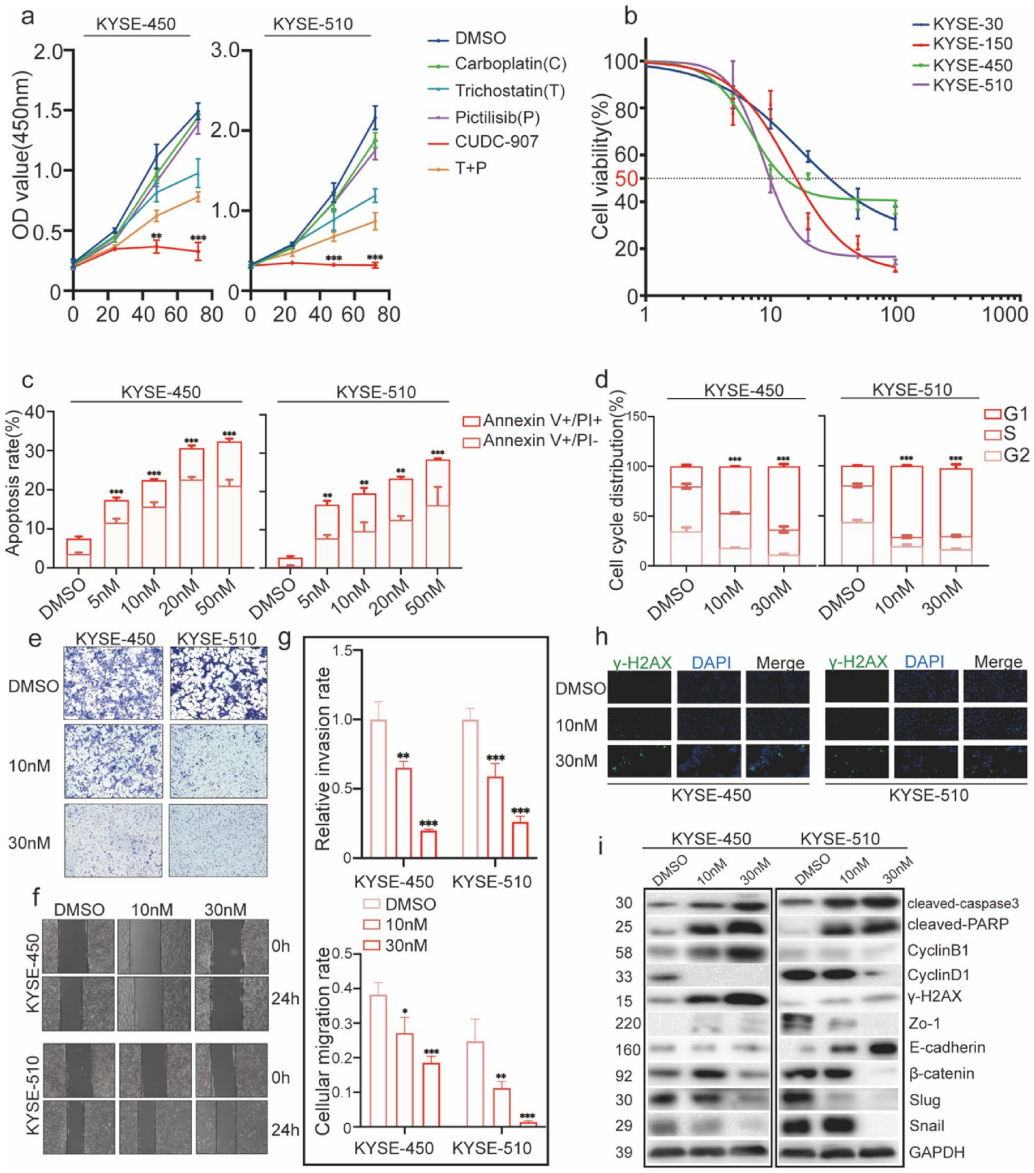
Anti-tumor ability of CUDC-907 in ESCC cell lines. **a** ESCC Cells were treated with various small molecule inhibitors (30 nM CUDC-907, trichostatin A, pictilisib, respectively), a common chemotherapy agent (3 μM carboplatin), and their combinations or DMSO (0 nM) in a time-dependent manner (0, 24, 48,72 h). Cell viability was confirmed using the CCK-8 experiment. **b** IC_50_ of different ESCC cells was calculated using GraphPad Prism. **c** Apoptosis rates were determined by Annexin V/PI assay after ESCC cells (KYSE-450 and KYSE-510) were exposed to CUDC-907 (5–50 nM) for 24 h. **d** Cell cycle profile analyses in ESCC cells (KYSE-450 and KYSE-510) exposed to CUDC-907 (10 and 30 nM) for 24 h. **e** Invasion assays were performed with ESCC cells after being exposed to CUDC-907 (10 and 30 nM) for 24 h by Transwell chambers. Scale bar: 100 μm. **f** Migration abilities were tested by a wound-healing assay in ESCC cells (KYSE-450 and KYSE-510) exposed to CUDC-907 (10 and 30 nM) for 24 h. Scale bar: 100 μm. **g** statistical analysis of migration assay and invasion assay. **h** Immunofluorescence analysis of γ-H2AX foci was performed in ESCC cells (KYSE-450 and KYSE-510) exposed to CUDC-907 (10 and 30 nM) for 24 h. Nuclei were visualized by using DAPI. Scale bar: 20 μm. **i** Levels of proteins related to the process of apoptosis, cell cycle, DNA damage, and epithelial–mesenchymal-transition (EMT) were measured via western blot analysis after ESCC cells (KYSE-450 and KYSE-510) were exposed to CUDC-907 (10 and 30 nM) for 24 h. Glyceraldehyde 3-phosphate dehydrogenase (GAPDH) was used as the loading control. Error bars are ± SD. *P < 0.05; **P < 0.01; ***P < 0.001

PI3K and HDAC inhibitors synergistically exert anti-tumor effects [21]. Thus, we compared the anti-proliferative effect of a common chemotherapy agent (carboplatin, 3 μM), various small-molecule-targeting inhibitors (30 nM CUDC-907, 30 nM trichostatin A, 30 nM pictilisib), and their combinations. CUDC-907 more effectively inhibited ESCC cell proliferation compared to carboplatin and other small-molecule-targeting inhibitors at the same dose (Fig. 2a). Based on the different expression of PI3K-Akt pathway-related proteins and HDACs in ESCC cell lines shown in Fig. 1c–e, the efficiency of inhibiting cell proliferation displayed by CUDC-907 was investigated in four different ESCC cell lines (KYSE-150, KYSE-450, KYSE-510, and KYSE-30). The results showed that CUDC-907 could inhibit cell proliferation significantly in a concentration- and time-dependent fashion (Additional file 1: Fig. S2a). According to the CCK-8 proliferation assay results at 48 h, cell viability was calculated to obtain the IC_50_ (half-maximal inhibitory concentration) values of different ESCC cell lines (Fig. 2b, Table 2). Then, we tested the alteration of PI3K-Akt pathways and HDACs in ESCC cell lines after CUDC-907 treatment to confirm the results above (Additional file 1: Fig. S3a, b).

**Table 2:** Half maximal inhibitory concentration (IC50) of CUDC-907 in different ESCC cell lines

ESCC cell lines	IC50 (nM)
KYSE-150	14.27 [95% CI (11.52–17.67)]
KYSE-450	6.893 [95% CI (6.303–7.537)]
KYSE-510	8.703 [95% CI (8.252–9.178)]
KYSE-30	16.95 [95% CI (12.80–22.46)]

*CI* confidence interval

To conclude the potential mechanism of how CUDC-907 inhibits cell proliferation, the effects of CUDC-907 treatment on apoptosis and cell cycle progression were tested by flow cytometry. We treated four different ESCC cell lines by CUDC-907 with doses ranging from 5 to 50 nM for 24 h and observed increased concentration-dependent apoptosis activity with Annexin V/PI assay (Fig. 2c, Additional file 1: Fig. S2b). We next evaluated the effect of CUDC-907 treatment on cell cycle progression by treating ESCC cell lines with doses of 10 and 30 nM. G2/M phase arrest was found after 12 h of CUDC-907 exposure (Fig. 2d, Additional file 1: Fig. S2c). Moreover, the key molecules involved in the cellular process of apoptosis and cell cycle were confirmed by western blot. The results showed significantly increased expression of cleaved-caspase3, cleaved-PARP, and cyclinB1, as well as downregulated expression of cyclinD1 in these ESCC cells (Fig. 2i, Additional file 1: Fig. S2h).

### Inhibition of cell migration and invasion by CUDC-907

A wound-healing assay and Transwell chamber assay were employed to verify the inhibition efficacy of CUDC-907 on the migration and invasion abilities of ESCC cells. We observed that CUDC-907 could significantly suppress cell migration in ESCC cells 24 h after scratching (Fig. 2f, Additional file 1: Fig. S2d). In the Transwell invasion assay, four kinds of ESCC cell lines were seeded in the upper chamber covered with Matrigel, and a decrease of invasion was found after CUDC-907 treatment (Fig. 2e, Additional file 1: Fig. S2e). We further evaluated the alterations of proteins related to cell migration and invasion. The outcomes suggested that CUDC-907 markedly upregulated E-cadherin and decreased Zo-1, β-catenin, Slug, and Snail in ESCC cells (Fig. 2i, Additional file 1: Fig. S2h).

### DNA damage in ESCC cells by CUDC-907

Previous reports [15, 16] indicated that CUDC-907 induces DNA damage in several cancer cell types. In this study, a known biomarker, γ-H2AX, was used to identify the occurrence of DNA damage via immunofluorescence assay after CUDC-907 treatment for 24 h. The exposure to CUDC-907 increased γ-H2AX foci formation in ESCC cells (Fig. 2h, Additional file 1: Fig. S2g). In addition, we detected the expression of γ-H2AX by western blot in these ESCC cells (Fig. 2i, Additional file 1: Fig. S2h).

### Autophagy induced by CUDC-907 in ESCC cell lines

**Fig. 3:**
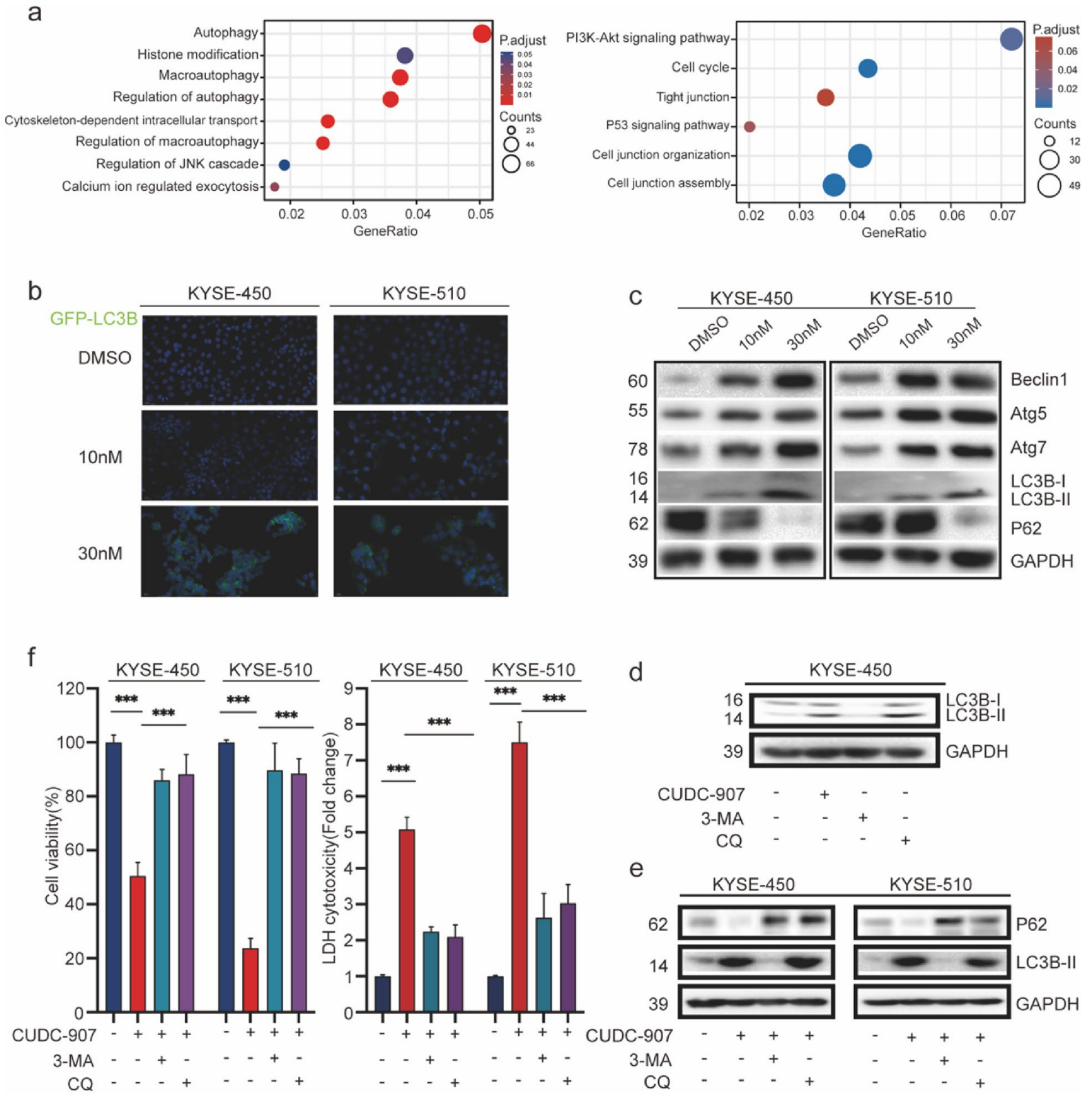
Autophagy induced by CUDC907 in ESCC cell lines and its regulation in autophagy flux. **a** Gene function enrichment analysis was performed using the Gene Ontology (GO) database after CUDC-907 treatment in ESCC cell lines. **b** Formation of autophagosomes showed by cellular fluorescence image. CUDC-907-treated ESCC cells (KYSE-450 and KYSE-510) obtained punctate profile of GFP-LC3B. Scale bar: 20 μm. **c** Autophagy-related protein expressions of Beclin-1, Atg5, Atg7, LC3B-I/II, and P62 were determined by western blot analysis. **d** Autophagic flux experiment was verified by western blot analysis using 3-MA (5 mM) or CQ (20 µM) in KYSE-450 cells. **e**, **f** Western blot analysis of P62 and LC3B-I/lC3-II. Cell viability and LDH release in CUDC-907 (30 nM, 48 h)-treated KYSE-450 and KYSE-510 cells, pretreated with 3-MA (5 mM) or CQ (20 μM) for 12 h. Error bars are ± SD. *P < 0.05; **P < 0.01; ***P < 0.001

To address the underlying molecular mechanism responsible for anti-tumor effects of CUDC-907 in ESCC cell lines, we performed RNA-sequencing analysis to compare the gene expression in DMSO-treated and CUDC-907-treated ESCC cells (KYSE-450 cell line). There were 974 downregulated genes and 872 upregulated genes in the CUDC-907-treated group after being exposed to 30 nM CUDC-907 for 24 h. As these genes were involved in various cell functions, we performed functional enrichment analysis using the Gene Ontology (GO) database. A comprehensive analysis combining the rich factor, Q value (adjusted P-value), and gene number showed that changes in the following cell functions were significantly enriched: autophagy (first), histone modification, macroautophagy, and regulation of autophagy (Fig. 3a). We further analyzed the downregulated genes and showed that the PI3K-Akt signaling pathway, cell cycle, and tight junction pathways were significantly inhibited (Fig. 3a). These data indicated that CUDC-907 could induce autophagy in ESCC cells, and autophagy-mediated cell death may be a key mechanism for its anti-tumor effects. To prove this hypothesis, we first performed a GFP-LC3 dot formation assay to confirm that autophagy in ESCC cells was induced by CUDC-907. The results showed that CUDC-907 treatment enhanced GFP-LC3 puncta generation in ESCC cells in a dose-dependent manner (Fig. 3b, Additional file 1: Fig. S4a). Furthermore, levels of autophagy-related proteins were determined using western blot analysis, and we found a significant increase in the expression of Atg5, Atg7, LC3B-II, and Beclin-1, while P62 expression was decreased (Fig. 3c).

Autophagic flux experiments were employed to testify the biological identity of autophagy in the anti-tumor effect of CUDC-907 using two kinds of classic autophagy inhibitors, 3-MA and CQ. 3-MA, a class III PI3K inhibitor, functions by blocking autophagosome formation in the early stage of autophagy. Unlike 3-MA, CQ plays a role in late-stage autophagy and works as an obstruction for autophagosomes to merge with lysosomes [22, 23]. Based on the IC_50_ value of these four ESCC cells, autophagic flux experiments using 3-MA (5 mM) and CQ (20 µM) were confirmed by western blot in KYSE-450 cells (Fig. 3d). To investigate whether CUDC-907 regulated cell death dependent on cytotoxic autophagy, ESCC cells were treated with CUDC-907 (30 nM) or not, after prior exposure to 3-MA (5 mM), CQ (20 µM) for 6 h. When exposed to 3-MA or CQ separately, cell viability and LDH release of ESCC cells presented no significant difference. However, autophagy inhibitors could drastically strengthen cell viability and alleviate LDH release to a distinct extent in CUDC-907-treated ESCC cells (Fig. 3f). We then evaluated p62 and LC3B-I/II levels via western blot analysis (Fig. 3e). Our results indicated that CUDC-907 regulates autophagic flux in ESCC cells.

mTOR plays an important role in regulating autophagy [24]. Because CUDC-907 could affect PI3K-Akt signal pathways, we first verified the effect of CUDC-907 on mTOR-mediated autophagy. The results showed that CUDC-907 could induce autophagy by inhibiting mTOR activation and promoting the activation of downstream ULK1. The use of mTOR inhibition or the rapamycin/mTOR activator MHY1485 could promote or reverse this process, respectively (Additional file 1: Fig. S4c). We also knocked down ULK1 expression in esophageal cancer cells and showed that the effect of CUDC-907-induced autophagy was weakened (Additional file 1: Fig. S4d). Similar results were observed in cell viability and LDH release assays (Additional file 1: Fig. S4e). These results demonstrated that CUDC-907 could promote autophagy and cell death by inhibiting mTOR activation.

### Regulation of CUDC-907-induced autophagic cell death in ESCC cell lines via ROS production via LCN2 inhibition

**Fig. 4:**
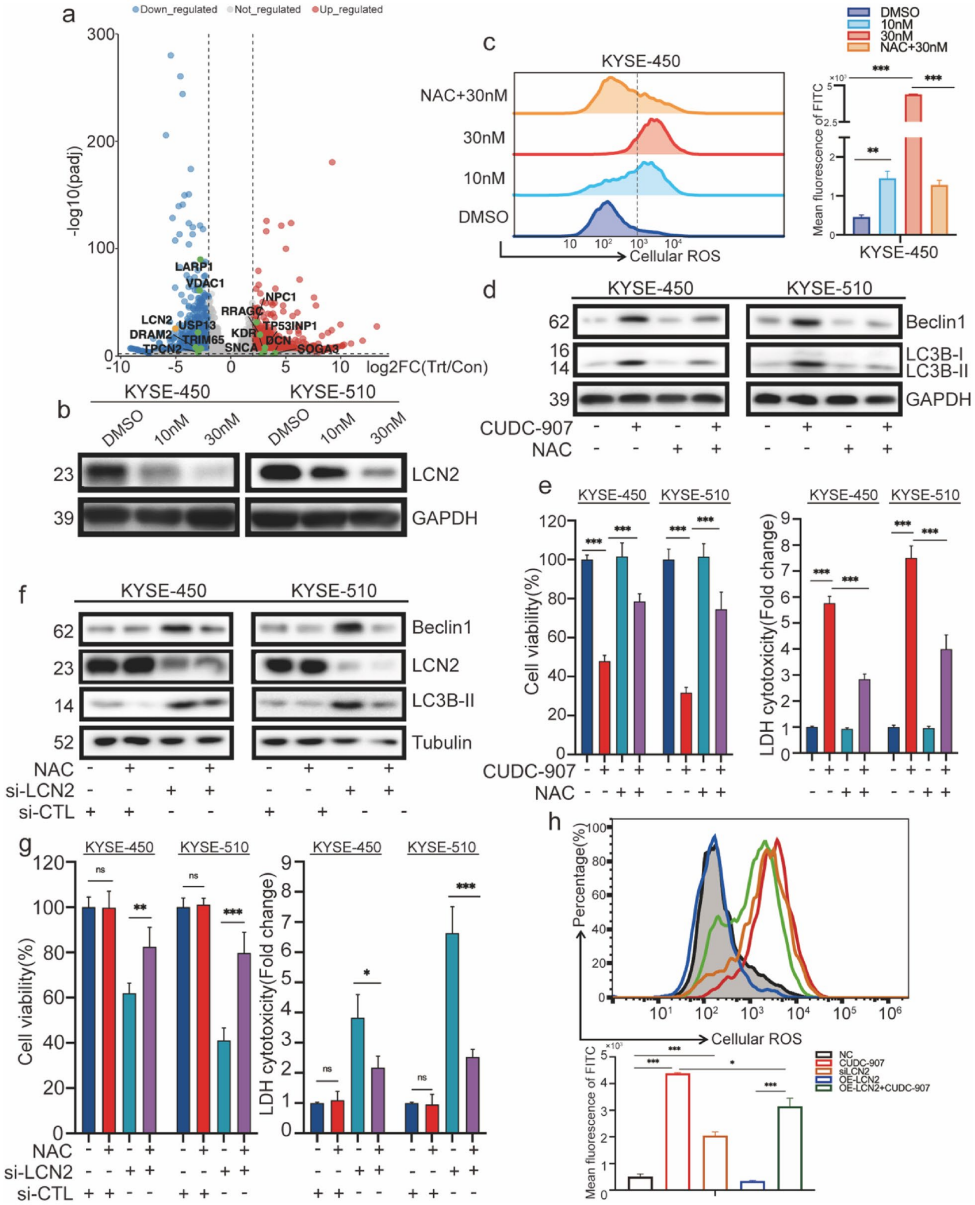
LCN2 inhibition regulating CUDC-907 induced autophagic cell death in ESCC cell lines via ROS production. **a** A volcano plot of the RNA-seq analysis shows that the expression of different genes changed after CUDC-907 treatment. **b** LCN2 protein expression was assayed using western blot analysis in KYSE-450 and KYSE-510 cells treated with DMSO, 10 or 30 nM CUDC-907. **c** Flow cytometry for the ROS generation assay in KYSE-450 cells treated with DMSO, 10 nM or 30 nM CUDC-907, or pretreated with NAC + 30 nM CUDC-907. **d**, **e** Western blot analysis of Beclin1 and LC3B-I/LC3B-II. Cell viability and LDH release in CUDC-907 (30 nM, 48 h)-treated KYSE-450 and KYSE-510 cells, pretreated with NAC (5 mM) or no treatment for 6 h. **f**, **g** Western blot analysis of Beclin1, LCN2, and LC3B-I/lC3-II. Cell viability and LDH release in CUDC-907 (30 nM, 48 h)-treated KYSE-450 and KYSE-510 cells, transfected with siLCN2 or negative control treatment for 48 h. **h** Flow cytometry as a measure of ROS levels in different types of treatment in KYSE-450 cells, histograms indicate the change in the mean of fluorescence in different groups of KYSE-450 cells. Error bars are ± SD. *P < 0.05; **P < 0.01; ***P < 0.001

To determine which molecule plays a major role in CUDC-907-induced cytotoxic autophagy in esophageal cancer cells, we selected several autophagy-related genes that were upregulated or downregulated based on the RNA-sequencing results. The gene lipocalin 2 (LCN2), which is related to iron transport balance and endoplasmic reticulum (ER) stress, was found to be most evidently downregulated (Fig. 4a). Previous studies reported [25, 26] that an imbalance of iron transport and a lack of reductive iron ions could lead to reactive oxygen species (ROS) accumulation in cells when LCN2 is downregulated, further aggravating ER stress. So we first checked cellular reductive iron and found CUDC-907 could reduce cellular iron levels (Additional file 1: Fig. S5b). To verify whether LCN2 was downregulated at the protein level in esophageal cancer cells, we treated cancer cells with different concentrations of CUDC-907 and performed western blot analysis, showing that LCN2 was considerably decreased at the protein level (Fig. 4b). To find out how LCN2 was downregulated by CUDC-907, we performed ChIP-qPCR assay of H3K9ac and H3K27ac in KYSE-450 cells and the results showed that CUDC-907 didn’t regulate the enrichment of H3K9ac and H3K27ac in LCN2 promotor region (Additional file 1: Fig. S5c). Except epigenetic regulation, recent research has demonstrated that CUDC-907 inhibited NF-κB activation, and NFκB1 and RELA/P65 transcriptionally regulated LCN2 (https://www.grnpedia.org/trrust/). Our western blot analysis also found that CUDC-907 could inhibit phosphorylation of IKKα, P65 and IKBα, and meanwhile downregulate the expression of LCN2 (Additional file 1: Fig. S5e).

ROS are reportedly involved in autophagy [27, 28]. We first determined the changes of cellular ROS in the KYSE-450 cells after treatment with different concentrations of CUDC-907 (DMSO, 10 nM, and 30 nM). Flow cytometry using the DCFH-DA probe detected an incremental ROS generation, which could be sharply attenuated by ROS scavenger NAC (Fig. 4c). Meanwhile, we found ROS scavenger NAC weakened the decrease in cell viability and increased LDH release (Fig. 4e) caused by CUDC-907 exposure. The protein levels of Beclin1 and LC3-I/II confirmed by western blot (Fig. 4d) agreed with the cell viability assay results. Hence, ROS are convincingly associated with cytotoxic autophagy caused by CUDC-907.

To confirm the relationship between LCN2 downregulation in ROS-induced autophagy and cell death, we constructed LCN2-knockdown cell lines and LCN2-overexpression cell lines and showed that ROS scavenger NAC weakened the decrease in cell viability and the increase in LDH release (Fig. 4g) caused by LCN2 knockdown. Beclin1 and LC3-I/II expression was considerably decreased in the NAC-treated group (Fig. 4f). In another ROS generation assay, LCN2 knockdown resulted in ROS accumulation, whereas LCN2 overexpression significantly decreased ROS levels compared to the control group after CUDC-907 treatment (Fig. 4h).

### ROS-IRE1α-JNK pathways related to CUDC-907-induced autophagic cell death

**Fig. 5:**
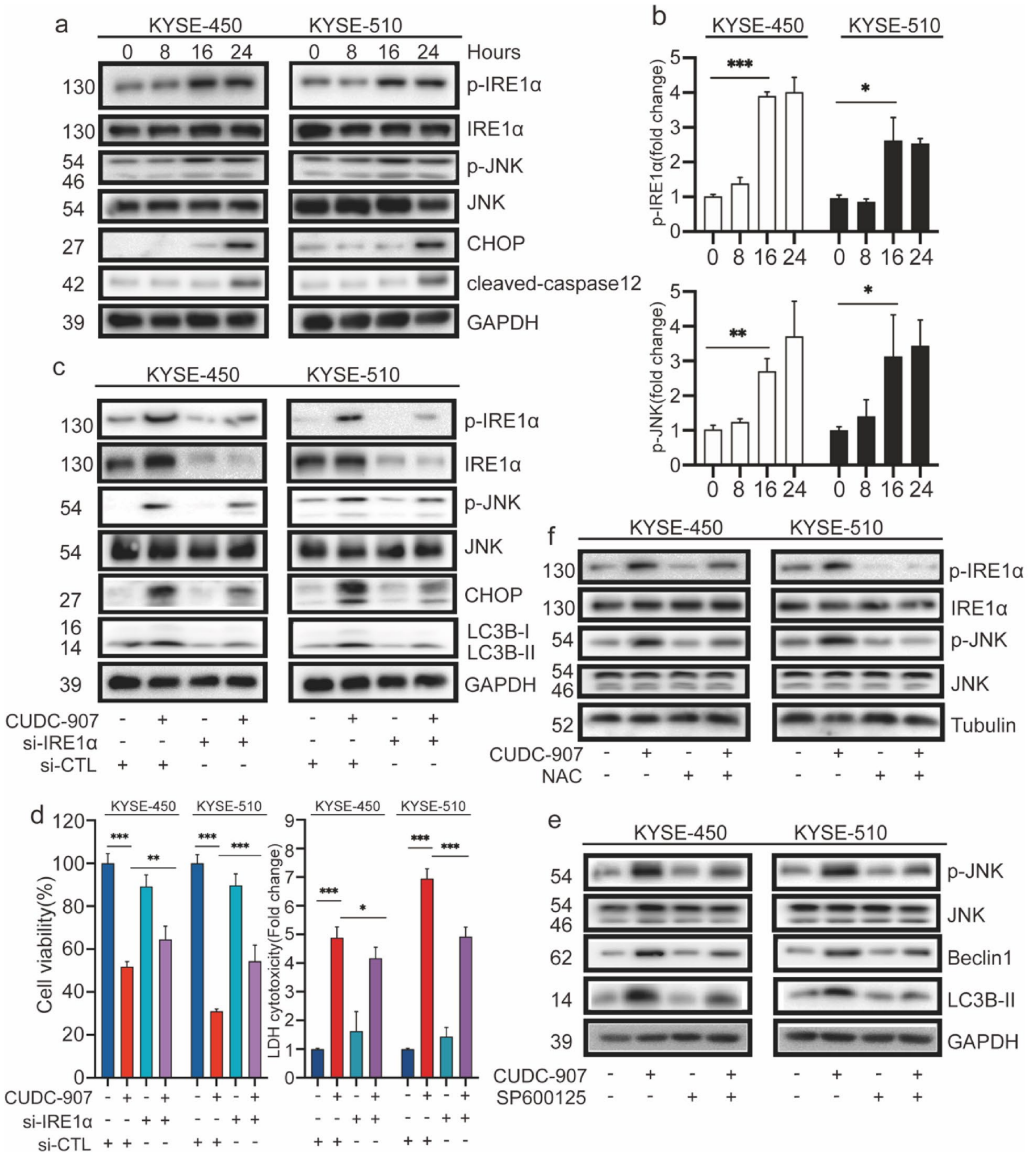
ROS-IRE1α-JNK pathways are related to autophagic cell death induced by CUDC-907. **a** Western blot analysis of p-IRE1α/IRE1α, p-JNK/JNK, CHOP, and cleaved caspase 12 in CUDC-907 (30 nM)-treated KYSE-450 and KYSE-510 cells in a time-dependent manner. **b** Ratio of p-IRE1α/GAPDH and p-JNK/GAPDH in CUDC-907-treated ESCC cells determined using Image J. **c**, **d** Western blot analysis of p-IRE1α/IRE1α, p-JNK/JNK, CHOP, and LC3B-I/lC3-II. Cell viability and LDH release of CUDC-907 (30 nM, 48 h)-treated KYSE-450 and KYSE-510 cells with siIRE1α or negative control treatment for 48 h. **e** Western blot analysis of p-JNK/JNK, Beclin1, and LC3B-I/lC3-II in CUDC-907 (30 nM, 48 h)-treated ESCC cells (KYSE-450 and KYSE-510) with SP600125 (10 μM) or not for 48 h. **f** Western blot analysis of p-IRE1α/IRE1α, p-JNK/JNK in CUDC-907 (30 nM, 48 h)-treated KYSE-450 and KYSE-510 cells, pretreated with NAC (5 mM) or no treatment for 6 h. Error bars are ± SD. *P < 0.05; **P < 0.01; ***P < 0.001

ER stress has been implicated in cellular autophagy and apoptosis [29]. To determine whether CUDC-907 induces ER stress, we examined the alteration of two main ER stress-related signaling pathways, including PERK-eIF2α and IRE1α-JNK. When we first monitored the PERK-eIF2α pathway in CUDC-907 (30 nM)-exposed esophageal cancer cells, no significant change of p-PERK and p-eIF2α was detected by western blot, suggesting the inactivation of the PERK-eIF2α signal cascade (Additional file 1: Fig. S6d). In contrast, the levels of p-IRE1α exhibited a time-dependent increase in response to CUDC-907 (30 nM) exposure. High levels of p-JNK, CHOP, and caspase12 were also triggered by CUDC-907, suggesting the activation of the IRE1α-JNK pathway (Fig. 5a, b). By transducing the ER stress signal from the cytosol to the nucleus (CHOP), the IRE1α-mediated signal cascade is believed to be critical for autophagy induced by ER stress in cancer. To gain a deeper understanding of how IRE1α influences CUDC-907-induced ER stress, ESCC cells were transfected with siRNA-IRE1α. The results showed that, compared to the CUDC-907-treated control group, IRE1α knockdown + CUDC-907 led to a significant rise in cell viability and correspondingly the decrease in LDH release (Fig. 5d). In addition, p-IRE1α, p-JNK, CHOP, and LC3-II were downregulated in the IRE1α knockdown + CUDC-907-treated group, as evidenced by western blot (Fig. 5c). JNK is another essential molecule in ER stress and other biological processes. To testify its role in ER stress induced by CUDC-907, SP600125, a JNK inhibitor, was applied in our study. The results showed that CUDC-907 regulates JNK activation in ESCC cells. Beclin1 and LC3B-II were considerably decreased by CUDC-907 + JNK inhibitor treatment than the CUDC-907 treatment alone, as confirmed by the western blot analysis (Fig. 5e). Subsequently, we evaluated the protein levels of p-IRE1α and p-JNK in CUDC-907 + NAC treatment compared to the CUDC-907 treatment alone (Fig. 5f). Overall, our results indicate that the blockage of the IRE1α-mediated signal cascade could suppress cytotoxic autophagy induced by CUDC-907 in ESCC cell lines. Besides, ROS accumulation is associated with the IRE1-JNK pathway activation. The functional enrichment analysis performed using the GO database also revealed the upregulation of the JNK cascade (Fig. 3a).

### Anti-tumor ability of CUDC-907 in vivo

**Fig. 6:**
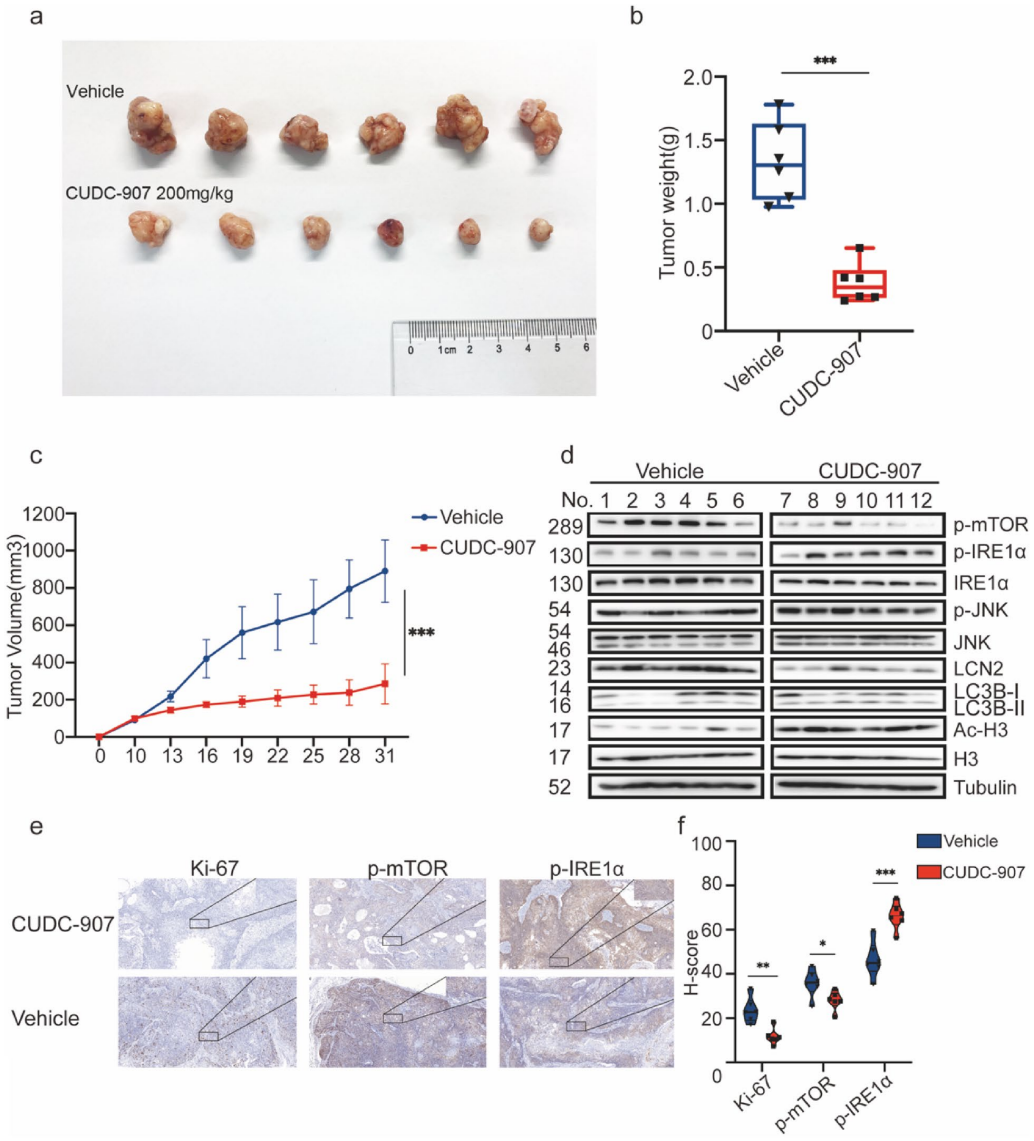
Anti-tumor ability of CUDC-907 in vivo. **a** On day 31, all mice were euthanized; primary tumor tissues were collected and imaged. **b** Weight of tumors derived from nude mice in the two groups. **c** Curve of tumor volume. **d** Expression levels in tumor tissues derived from two groups of nude mice were determined by western blot analysis to test the alteration of p-mTOR, p-IRE1α, LCN2, LC3B, and Ac-H3. **e** Representative images of Ki-67, p-mTOR, and p-IRE1α stained by immunohistochemical methods in control and treatment groups. **f** H-score methods were applied to evaluate immunohistochemical staining levels. Scale bar: 20 μm and 100 μm. Error bars are ± SD. *P < 0.05; **P < 0.01; ***P < 0.001

The in vivo anti-tumor efficacy of CUDC-907, as illustrated in Fig. 6a, was investigated in a subcutaneous xenograft model of human esophageal cancer cell line KYSE-450 in immune-deficient nude mice. The 200 mg/kg CUDC-907-treated group presented less overall tumor burden, including tumor weight and volume in mouse models, than the control group (Fig. 6b, c) on days 25 and 31 after drug administration (Additional file 1: Fig. S6e). No significant difference was observed in the bodyweight of mice (Additional file 1: Fig. S6f). The results of the western blot analysis showed that CUDC-907 inhibited HDACs (as indicated by the accumulation of Ac-H3 and PI3K (as indicated by the decrease in p-mTOR) (Fig. 6d). We also demonstrated the activation of the IRE1-JNK signaling pathway and a decrease in LCN2, which were strongly related to the CUDC-907-induced autophagy, confirmed by the increase in LC3B (Fig. 6d). The IHC assay showed a decrease in Ki-67 and p-mTOR levels and accumulation of IRE1 in the CUDC-907-treated group (Fig. 6e, f).

## Discussion

**Fig. 7:**
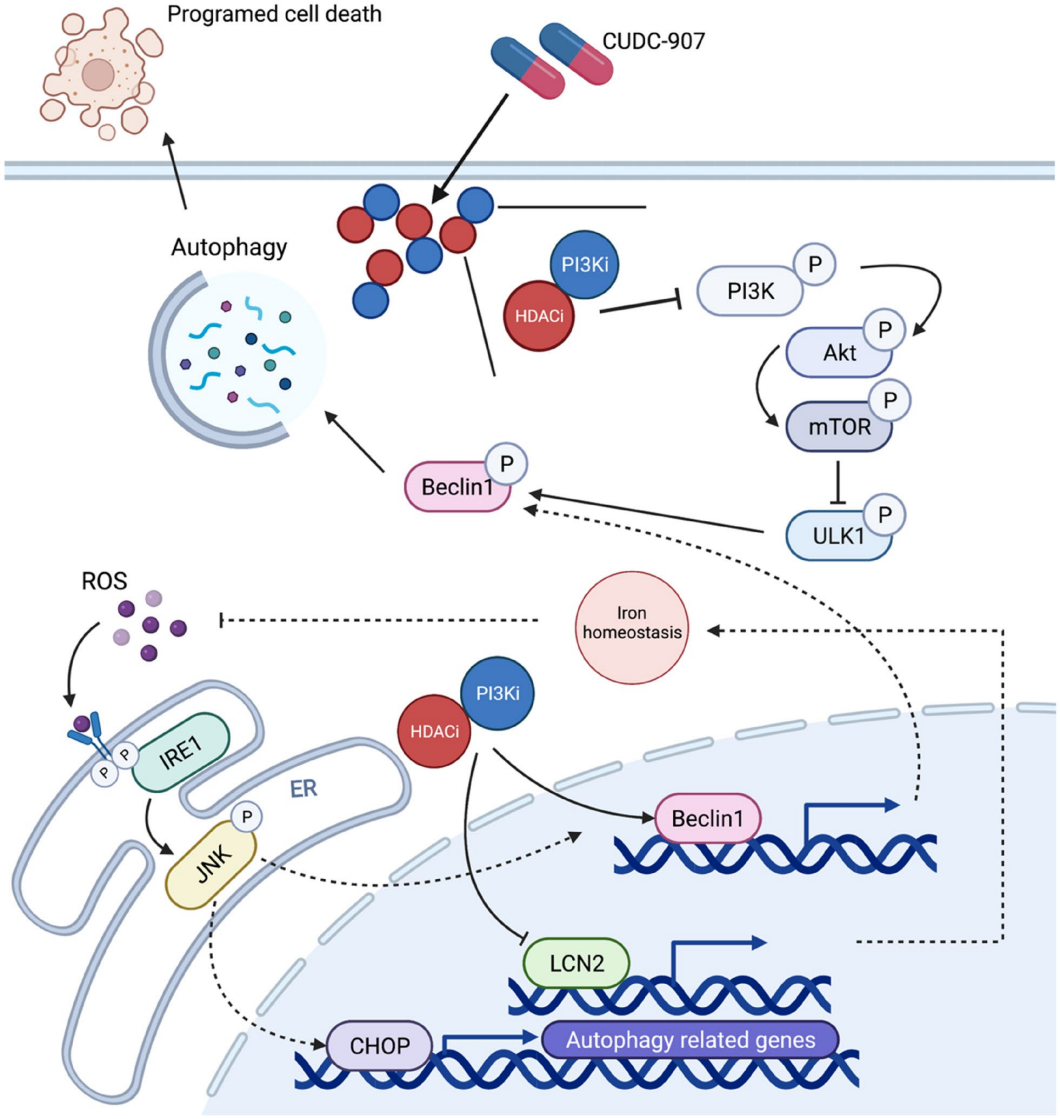
Working model for the mechanism of anti-tumor effect on CUDC-907. A working model showing how dual-inhibitor CUDC-907 activates cellular autophagy by inhibiting mTOR or downregulating LCN2 to accumulate ROS, thus, activating the IRE1α-JNK-CHOP cascade

In this article, we found that PI3K-Akt pathways and HDACs were highly activated in ESCC patients and esophageal cancer cell lines. Furthermore, CUDC-907 could inhibit multiple biological processes, including proliferation, migration, and survival in various esophageal cancer cell lines. More importantly, CUDC-907 could downregulate LCN2 expression in esophageal cancer cell lines, leading to ROS accumulation, ultimately activating cytotoxic autophagy through ER stress pathway, followed by programmed cell death. At the end of this article, we confirmed the in vitro findings in a human esophageal cancer KYSE-450 xenograft nude mouse model. A working model of our study has been summarized (Fig. 7). Our results show that CUDC-907 can be used as a new type of targeted drug for esophageal cancer therapy.

To date, simultaneous inhibition of phosphoinositide 3-kinase (PI3K) and histone deacetylases (HDACs) is regarded as more effective in the cure for lymphoma or other solid tumors than a single PI3K or HDAC inhibitor [15, 17, 30]. In addition, hybrid molecules such as CUDC-907 do not readily diffuse out of cells after being transformed into their pharmacologically active forms. Thus, their intracellular concentrations can easily increase [21]. Hence, the de novo artificially synthesized drug CUDC-907, a dual-inhibitor of PI3K-Akt and HDACs, has gained research interest and was well characterized in our research. To comprehend the underlying theory of how CUDC-907 suppressed cell proliferation, migration, and invasion, we treated esophageal cancer cells with CUDC-907 at different concentrations. We found increased concentration-dependent apoptosis and G2/M phase arrest. Furthermore, expression levels of key molecular proteins related to viability, proliferation, migration, and invasion were significantly altered. We also explored the effect of CUDC-907 on tumor cells at the transcriptional level and found that CUDC-907-induced cytotoxic autophagy played an important role in promoting tumor cell death.

Autophagy, a common biological phenomenon that removes and clears impaired or redundant organelles, is also believed to be related to the occurrence of cancer and cancer cell sensitivity to treatment [31]. Autophagy plays a dual role in cancer owing to different cell types and genetic factors [23]. On the one hand, autophagy can improve cancer survival by reducing genomic DNA damage and limiting cell death under metabolic stress conditions and stimulant medications [27, 32]. However, on the other hand, cytotoxic autophagy is reportedly a new therapeutic strategy for treatment-insensitive cancers [33–35]. So far, there are plenty of chemicals or natural products used as drugs to activate the cytotoxic autophagy in cancer cells. For example, Kaempferol, a kind of potential HDAC inhibitor, mediates autophagy in gastric cancer cells by inhibiting G9a and stimulating ER stress, leading to IRE1-JNK-CHOP pathway activation [36]. Our results showed that autophagy of esophageal cancer cells was induced by CUDC-907 and the increase of LC3 transformation rate proved the existence of autophagy. In addition, both 3-MA and CQ autophagy inhibitors attenuated CUDC-907-induced cell death, suggesting that CUDC-907-induced cytotoxic autophagy is involved in cell death.

Lipocalin 2 (LCN2) is an oncogene that has been widely studied in the field of cancer [37–39] and cellular autophagy research [40, 41]. For example, the relatively poor prognosis of several invasive breast cancer [37], pancreatic cancer [42], and colorectal carcinoma [43] is closely related to LCN2 overexpression. In breast cancer, increased LCN2 levels promote cancer cell proliferation and angiogenesis [44]. The role of LCN2 in esophageal cancer has also been widely studied [38, 45]. LCN2, which is methylated at low levels but highly expressed in esophageal cancer, regulates the migration and invasion of esophageal cancer via multiple pathways [38]. Some reports have shown that LCN2 promotes migration and invasion through the ERK1/2 pathway [39]. Our results agreed with these reports; we observed that CUDC-907 could downregulate the ERK1/2 pathway and LCN2 expression, although the correlation between these two phenomena requires further investigation. In addition, LCN2 is also crucial in iron homeostasis, and the intracellular level of iron is closely related to ROS production [25], which may be a way to activate cellular autophagy. Herein we proved this hypothesis and found that CUDC-907 displayed anti-tumor effects by downregulating LCN2 to activate ROS-IRE1α-JNK mediated autophagy.

ROS regulates cancer cell survival [46]. Low concentrations of ROS are involved in cellular signal transduction, whereas excessive ROS can damage proteins and DNA, resulting in autophagy or cell death [46–48]. The present results showed that CUDC-907-induced ROS could significantly increase the level of autophagy markers; however, NAC pretreatment could significantly reverse CUDC-907-induced autophagy and cell death, suggesting that CUDC-907 plays an essential role in cell autophagy and death through ROS production. Moreover, ROS is one of the secondary messengers and plays a biological role by activating downstream molecules, particularly in the ER stress process [47, 49, 50]. Our experimental results suggested that CUDC-907 promoted autophagic death of esophageal cancer cells through ER stress. There are several signal cascades involved in ER stress. PERK-eIF2α and IRE1α-JNK are two classic signal cascades, so we screened these two endoplasmic stress-related signal cascades. When monitoring the PERK-eIF2α signal pathway in esophageal cancer cells after CUDC-907 treatment, we did not detect significant changes of p-PERK and p-eIF2α, suggesting the PERK signal cascade was inactivated. Nevertheless, the expression of p-IRE1α, p-JNK, CHOP, and caspase-12 were upregulated, indicating that the activation of CUDC-907-induced ER stress was mediated by the IRE1α-JNK signal cascade. To the best of our knowledge, JNK can induce autophagic death. For instance, JNK1 induces the phosphorylation of B-cell CLL/lymphoma 2 apoptosis regulator (Bcl-2) and incentivizes cytotoxic autophagy by destroying the Bcl-2-Beclin-1complex [33]. Meanwhile, CHOP, as an important downstream player in the JNK cascade, is vital to cell death induced by ER stress through PERK and IRE1α signaling pathways [36, 51]. Interestingly, we found that inhibition of IRE1α led to a decrease of CHOP, indicating that the upstream signal of CHOP reduced autophagic death by downregulating CHOP. These data suggested that CUDC-907 could induce cytotoxic autophagy and cell death in esophageal cancer cells through IRE1α-JNK signal cascade, whereas IRE1α gene knockout could block CUDC-907-treated autophagic death.

## Conclusions

In summary, the results of this study showed that CUDC-907 could promote the anti-tumor effect by inhibiting the proliferation, invasion, and migration of esophageal cancer cells. Furthermore, this drug could downregulate LCN2 expression in esophageal cancer cells and increase the accumulation of intracellular ROS levels to activate the ER stress response, resulting in a significant increase in autophagy, followed by tumor cell death. These results were confirmed in the human esophageal cancer KYSE-450 xenograft nude mouse model. Our data indicate that CUDC-907 is an achievable and efficient medication for treating esophageal cancer, which warrants further clinical investigation. Although we have presented a comprehensive characterization of how LCN2 downregulation activates autophagy in CUDC-907-treated ESCC cells, future research should focus on how CUDC-907 downregulates LCN2 expression. A more in-depth understanding of the anti-tumor effect of CUDC-907 in esophageal cancer will benefit the clinically targeted treatment of ESCC.

## Supplementary Information

**Additional file 1: Figure S1. a.** Clinical characteristics were summarized in Table S1 **b.** Representative computerized tomography (CT) images from one of patient with esophageal cancer before and after surgery were presented. **Figure S2. a**. ESCC cell lines were treated with variable concentrations of CUDC‐907 in 96‐well plates in a time-dependent manner. The CCK-8 assay was performed to determine viable cells. **b.** Apoptosis rates were determined by Annexin V/PI assay after ESCC cells were exposed to CUDC-907 (5 ~ 50 nM) for 24 h. **c.** Cell cycle profile analysis in ESCC cells exposed to CUDC-907(10 and 30 nM) for 24 h. **d.** Migration assays were tested by a wound healing assay in ESCC cells (KYSE30 and KYSE150) exposed to CUDC-907(10 and 30 nM) for 24 h. Scale bar: 100 μm. **e.** Invasion assays were performed with ESCC cells after being exposed to CUDC-907(10 and 30 nM) for 24 h by Transwell chambers. Scale bar: 100 μm. **f.** statistical analysis of migration assay and invasion assay. **g.** Immunofluorescence analysis of γ-H2AX foci were performed in in ESCC cells (KYSE30 and KYSE150) exposed to CUDC-907(10 and 30 nM) for 24 h. Nuclei were visualized by using DAPI. Scale bar: 20 μm. **h.** Levels of proteins related to process of apoptosis, cell cycle, DNA damage, and epithelial–mesenchymal-transition (EMT) were measured via western blot analysis after ESCC cells (KYSE30 and KYSE150) exposed to CUDC-907(10 and 30 nM) for 24 h. Glyceraldehyde 3-phosphate dehydrogenase (GAPDH) was used as loading control. Error bars are ± SD. *, P < 0.05; **, P < 0.01; ***, P < 0.001. **Figure S3. a, b, c.** Protein levels associated with PI3K-Akt pathway and HDACs were evaluated via western blot after ESCC cells were exposed to CUDC-907 (10 nM and 30 nM) for 24 h. Tubulin and GAPDH were employed as loading control. **Figure S4. a**.Formation of autophagosomes showed by cellular fluorescence image. CUDC-907-etreated ESCC cells (KYSE-30 and KYSE-150) obtained punctate profile of GFP-LC3B. **b.** Autophagy-related protein expressions of Beclin-1, Atg5, Atg7, Atg12, LC3B-I/II, and P62 were determined by western blot analysis. **c.** Western blot analysis of p-mTOR/mTOR, p-ULK1/ULK1, Beclin1, Atg5, Atg7, and LC3B-I/lC3-II in CUDC-907 (30 nM, 48 h)-treated KYSE-450 and KYSE-510 cells with rapamycin (5 nM) or MHY1485 (10 μM) treatment for 48 h. **d.** Western blot analysis of p-ULK1/ULK1, Beclin1, and LC3B-I/lC3-II in CUDC-907 (30 nM, 48 h)-treated KYSE-450 and KYSE-510 cells with siULK1 or negative control treatment for 48 h. **e. **Analysis of cell viability and LDH release were done by CCK-8 in CUDC-907 (30 nM, 48 h)-treated KYSE-450 and KYSE-510 cells with rapamycin or MHY1485 treatment for 48 h. Error bars are ± SD. *, P < 0.05; **, P < 0.01; ***, P < 0.001. **Figure S5. a.** Heatmap of selective seven upregulated and seven downregulated genes in ESCC cells. **b.** Intracellular iron levels in KYSE-450 and KYSE-510 after CUDC-907 treatment. **c.** ChIP-qPCR assay to reveal whether down-expressed LCN2 was regulated by H3 acetylation after CUDC-907 treatment. **d.** An online prediction website to predict LCN2 potential transcriptional factors (https://www.grnpedia.org/trrust/). **e.** Protein levels associated with NF-κB pathway were evaluated via western blot after ESCC cells were exposed to CUDC-907 (10 and 30 nM). Error bars are ± SD. *, P < 0.05; **, P < 0.01; ***, P < 0.001. **Figure S6.a, c**. The expression levels of IRE1α and LCN2 in KYSE-450 and KYSE-510 cells with siLCN2 or siIREα and negative control treatment for 48 h were confirmed by western blot. **b. **Western blot analysis of LCN2 in KYSE-450 and KYSE-510 cells with OE-LCN2 and negative control treatment for 48 h. **d.** Western blot analysis of expression of p-PERK/PERK and p-eIF2α/ eIF2α. **e.** Treatment schema. The vehicle group (N = 6) worked as control group and these mice were fed with 30% Captisol, whereas the treatment group (N = 6), which was 200 mg/kg CUDC-907 group, were fed with 200 mg/kg CUDC-907 dissolving in 30% Captisol. Dose regimen was followed by 5-days-on and 2-days-off via oral gavagea and started on day 10. **f.** Tumor growth inhibition upon CUDC-907 treatment in KYSE-450 ESCC xenografts. Curve of mice body weight were presented. Additional materials S1 and S2.

## Data Availability

The datasets used and analysed during the current study are available from the corresponding author on reasonable request.

## Abbreviations

PI3K: Phosphoinositide 3-kinase (PI3K)-protein kinase B
HDAC: Histone deacetylase
LCN2: Lipocalin 2
ER: Endoplasmic reticulum
CDX: Cell-derived xenografts
ESCC: Esophageal squamous cell carcinoma
siRNA: Small interfering RNA
OE: Overexpression
DMSO: Dimethyl sulfoxide
FBS: Fetal bovine serum
CQ: Chloroquine
3-MA: 3-Methyladenine
CCK-8: Cell counting kit-8
IC_50_: Half-maximal inhibitory concentration
LDH: Lactate dehydrogenase
IHC: Immunohistochemical/immunohistochemistry
CST: Cell signaling technology
DCFH-DA: Dichloro-dihydro-fluorescein diacetate
TCGA: The Cancer Genome Atlas
SD: Standard deviation
CI: Confidence interval
