# Supplementary material for: Anti-tumor effects of dual PI3K-HDAC inhibitor CUDC-907 on activation of ROS-IRE1α-JNK-mediated cytotoxic autophagy in esophageal cancer
Source: Cell Biosci. 2022 Aug 21;12:135. doi: 10.1186/s13578-022-00855-x (PMC9394063; doi:10.1186/s13578-022-00855-x)
Supplement: Supplementary file 1 — Additional file 1: Figure S1. a. Clinical characteristics were summarized in Table S1 b. Representative computerized tomography (CT) images from one of patient with esophageal cancer before and after surgery were presented. Figure S2. a. ESCC cell lines were treated with variable concentrations of CUDC‐907 in 96‐well plates in a time-dependent manner. The CCK-8 assay was performed to determine viable cells. b. Apoptosis rates were determined by Annexin V/PI assay after ESCC cells were exposed to CUDC-907 (5 ~ 50 nM) for 24 h. c. Cell cycle profile analysis in ESCC cells exposed to CUDC-907(10 and 30 nM) for 24 h. d. Migration assays were tested by a wound healing assay in ESCC cells (KYSE30 and KYSE150) exposed to CUDC-907(10 and 30 nM) for 24 h. Scale bar: 100 μm. e. Invasion assays were performed with ESCC cells after being exposed to CUDC-907(10 and 30 nM) for 24 h by Transwell chambers. Scale bar: 100 μm. f. statistical analysis of migration assay and invasion assay. g. Immunofluorescence analysis of γ-H2AX foci were performed in in ESCC cells (KYSE30 and KYSE150) exposed to CUDC-907(10 and 30 nM) for 24 h. Nuclei were visualized by using DAPI. Scale bar: 20 μm. h. Levels of proteins related to process of apoptosis, cell cycle, DNA damage, and epithelial–mesenchymal-transition (EMT) were measured via western blot analysis after ESCC cells (KYSE30 and KYSE150) exposed to CUDC-907(10 and 30 nM) for 24 h. Glyceraldehyde 3-phosphate dehydrogenase (GAPDH) was used as loading control. Error bars are ± SD. *, P < 0.05; **, P < 0.01; ***, P < 0.001. Figure S3. a, b, c. Protein levels associated with PI3K-Akt pathway and HDACs were evaluated via western blot after ESCC cells were exposed to CUDC-907 (10 nM and 30 nM) for 24 h. Tubulin and GAPDH were employed as loading control. Figure S4. a.Formation of autophagosomes showed by cellular fluorescence image. CUDC-907-etreated ESCC cells (KYSE-30 and KYSE-150) obtained punctate profile of GFP-LC3B. b. Autophagy-related prot [file 13578_2022_855_MOESM1_ESM.docx]

**Supplementary Figures:**

**Supplementary Figure 1.**

**
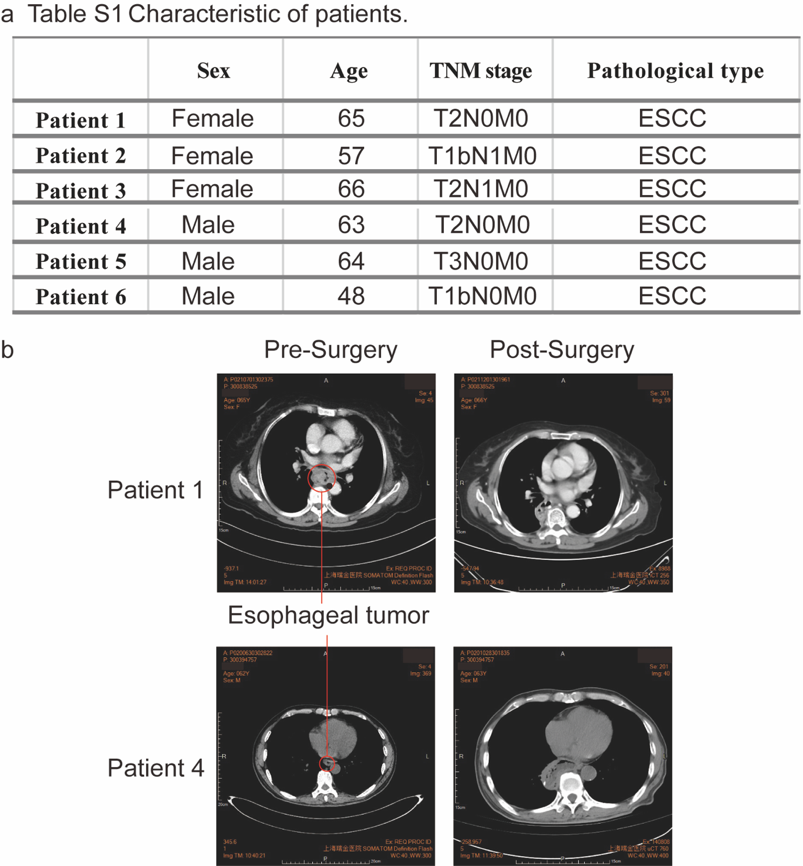
**

**Figure S1. a.** Clinical characteristics were summarized in Table S1 **b.** Representative computerized tomography (CT) images from one of patient with esophageal cancer before and after surgery were presented.

**Supplementary Figure 2.**

**
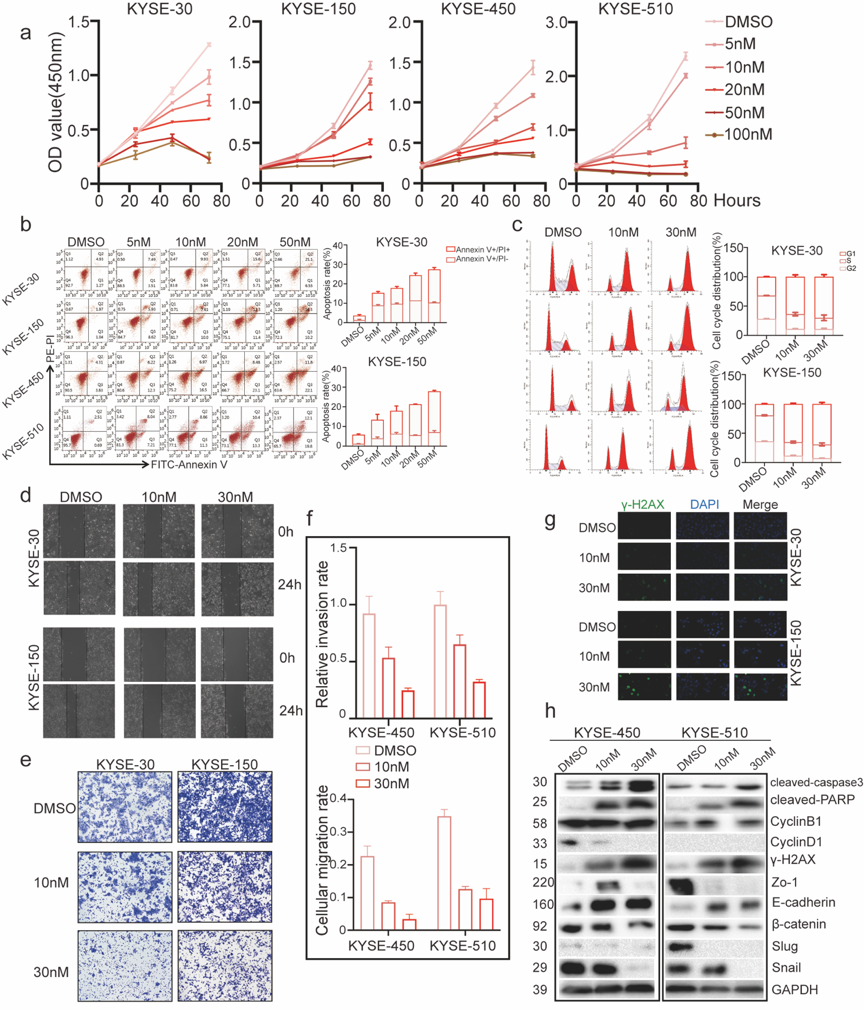
**

**Figure S2. a**. ESCC cell lines were treated with variable concentrations of CUDC‐907 in 96‐well plates in a time-dependent manner. The CCK-8 assay was performed to determine viable cells. **b.** Apoptosis rates were determined by Annexin V/PI assay after ESCC cells were exposed to CUDC-907 (5~50nM) for 24h. **c.** Cell cycle profile analyses in ESCC cells exposed to CUDC-907(10 and 30nM) for 24 h. **d.** Migration assays were tested by a wound healing assay in ESCC cells (KYSE30 and KYSE150) exposed to CUDC-907(10 and 30nM) for 24 h. Scale bar: 100μm. **e.** Invasion assays were performed with ESCC cells after being exposed to CUDC-907(10 and 30nM) for 24 h by Transwell chambers. Scale bar: 100μm. **f.** statistical analysis of migration assay and invasion assay. **g.** Immunofluorescence analysis of γ-H2AX foci were performed in in ESCC cells (KYSE30 and KYSE150) exposed to CUDC-907(10 and 30nM) for 24 h. Nuclei were visualized by using DAPI. Scale bar: 20 μm. **h.** Levels of proteins related to process of apoptosis, cell cycle, DNA damage, and epithelial–mesenchymal-transition (EMT) were measured via western blot analysis after ESCC cells (KYSE30 and KYSE150) exposed to CUDC-907(10 and 30nM) for 24 h. Glyceraldehyde 3-phosphate dehydrogenase (GAPDH) was used as loading control. Error bars are ± SD. *, P < 0.05; **, P < 0.01; ***, P < 0.001.

**Supplementary Figure 3.**


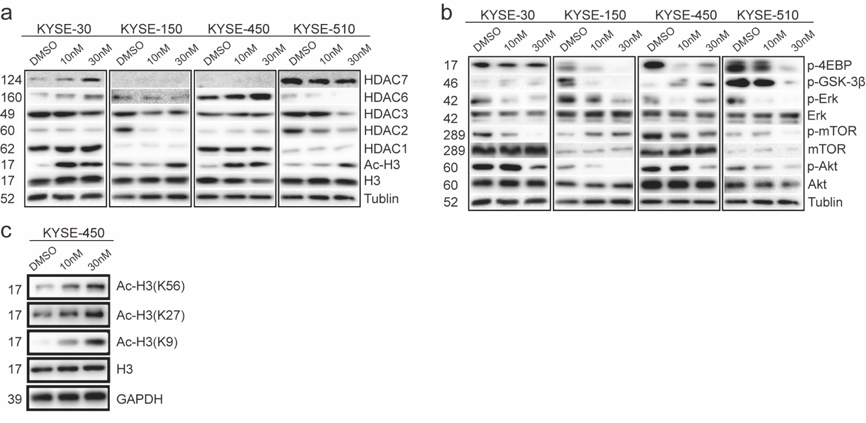


**Figure S3. a, b, c.** Protein levels associated with PI3K-Akt pathway and HDACs were evaluated via western blot after ESCC cells were exposed to CUDC-907 (10nM and 30nM) for 24h. Tubulin and GAPDH were employed as loading control.

**Supplementary Figure 4.**


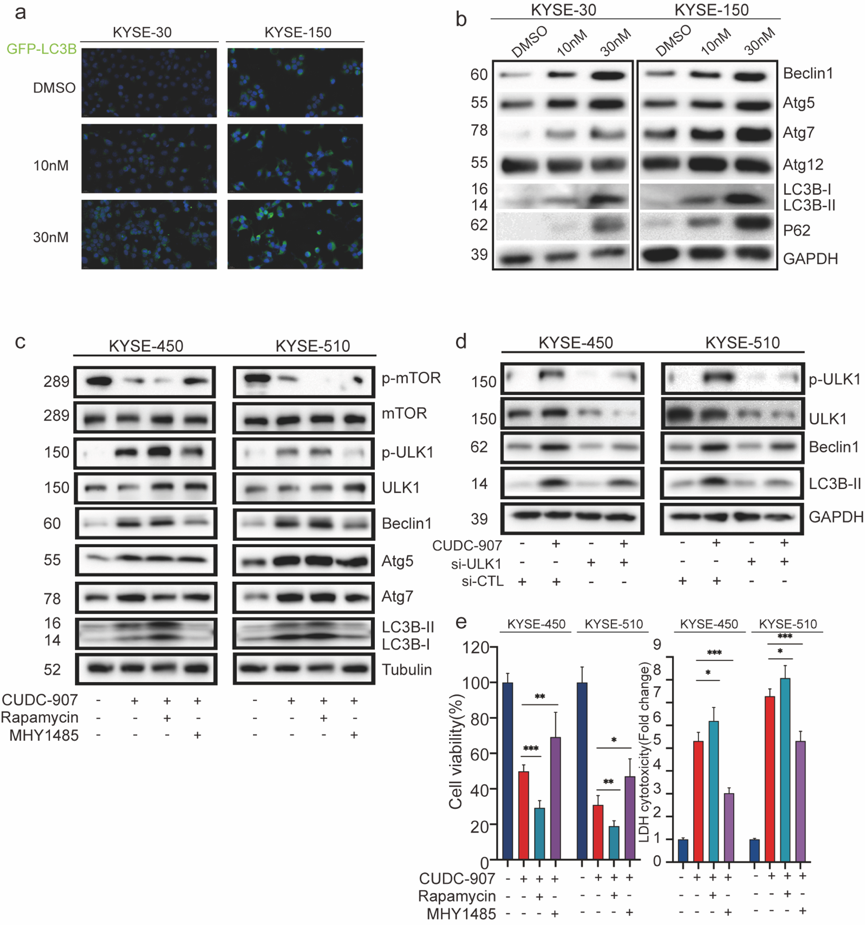


**Figure S4. a**. **b.** Formation of autophagosomes showed by cellular fluorescence image. CUDC-907-etreated ESCC cells (KYSE-450 and KYSE-510) obtained punctate proﬁle of GFP-LC3B. **c.** Autophagy-related protein expressions of Beclin-1, Atg5, Atg7, Atg12, LC3B-I/II, and P62 were determined by western blot analysis. **c.** Western blot analysis of p-mTOR/mTOR, p-ULK1/ULK1, Beclin1, Atg5, Atg7, and LC3B-I/lC3-II in CUDC-907 (30 nM, 48 h)-treated KYSE-450 and KYSE-510 cells with rapamycin (5 nM) or MHY1485 (10 μM) treatment for 48 h. **d.** Analysis of cell viability and LDH release were done by CCK-8 in CUDC-907 (30 nM, 48 h)-treated KYSE-450 and KYSE-510 cells with rapamycin or MHY1485 treatment for 48 h. **e.** Western blot analysis of p-ULK1/ULK1, Beclin1, and LC3B-I/lC3-II in CUDC-907 (30 nM, 48 h)-treated KYSE-450 and KYSE-510 cells with siULK1 or negative control treatment for 48 h. Error bars are ± SD. *, P < 0.05; **, P < 0.01; ***, P < 0.001.

**Supplementary Figure 5.**

**
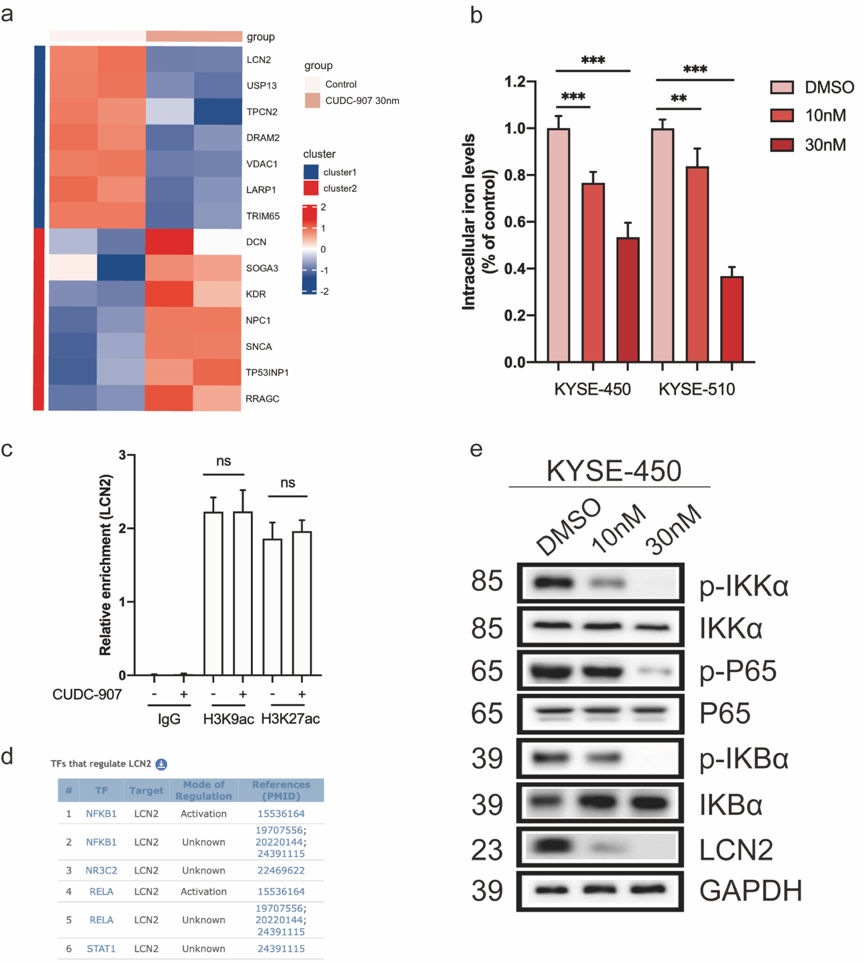
**

**Figure S5. a.** Heatmap of selective seven upregulated and seven downregulated genes in ESCC cells. **b.** Intracellular iron levels in KYSE-450 and KYSE-510 after CUDC-907 treatment. **c.** ChIP-qPCR assay to reveal whether down-expressed LCN2 was regulated by H3 acetylation after CUDC-907 treatment. **d.** An online prediction website to predict LCN2 potential transcriptional factors (<https://www.grnpedia.org/trrust/>). **e.** Protein levels associated with NF-κB pathway were evaluated via western blot after ESCC cells were exposed to CUDC-907 (10nM and 30nM). Error bars are ± SD. *, P < 0.05; **, P < 0.01; ***, P < 0.001.

**Supplementary Figure 6.**


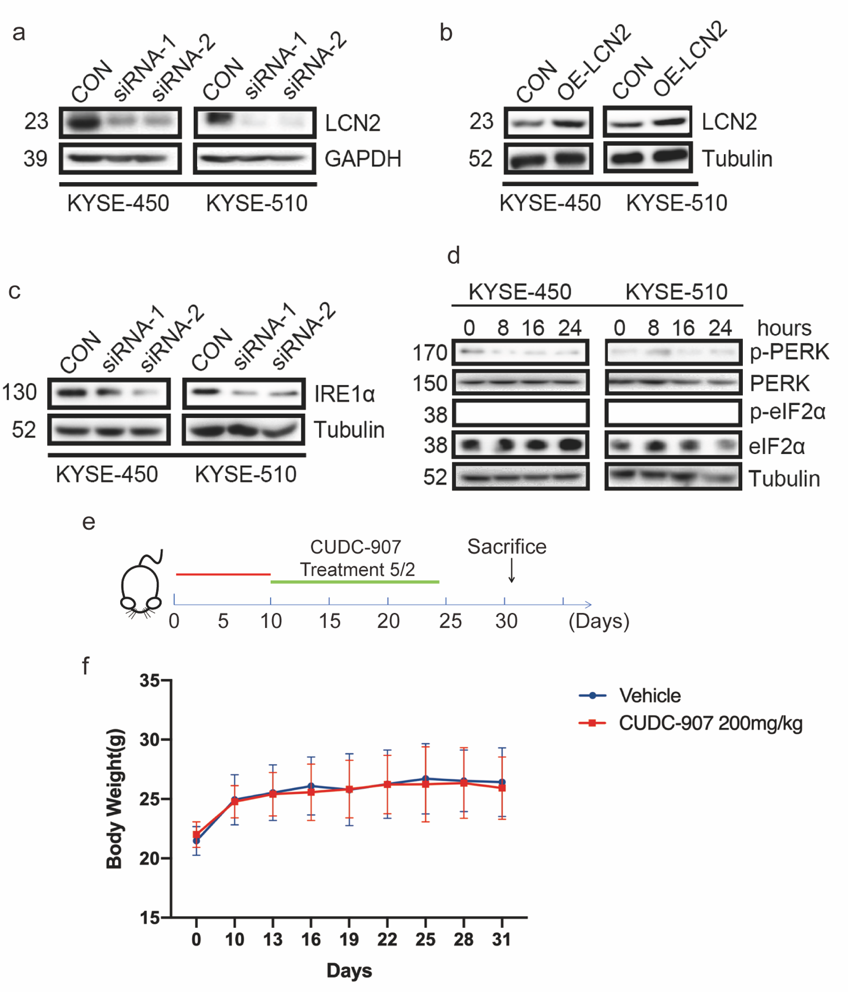


**Figure S6. a.** Western blot analysis of LCN2 in KYSE-450 and KYSE-510 cells with OE-LCN2 and negative control treatment for 48 h. **b, c**. The expression levels of IRE1α and LCN2 in KYSE-450 and KYSE-510 cells with IRE1α or LCN2 and negative control treatment for 48 h were confirmed by western blot. **d.** Western blot analysis of expression of p-PERK/PERK and p-eIF2α/ eIF2α. **e.** Treatment schema. The vehicle group (N=6) worked as control group and these mice were feed with 30% Captisol, whereas the treatment group (N=6), which means 200mg/kg CUDC-907 group, were feed with 200mg/kg CUDC-907 dissolving in 30% Captisol. Dose regimen was followed by 5-days on and 2-days-off via oral gavagea and started on day 10. CUDC-907 was administered via oral gavage (5/2, 200 mg/kg). **f.** Tumor growth inhibition upon CUDC-907 treatment in KYSE-450 ESCC xenografts. Curve of mice body weight were presented.

**Supplementary Materials**

**Supplementary Material 1:**

**Primary antibodies:**

The following antibodies were purchased from CST: Akt (#4691), p-Akt (Ser473)(#4060), mTOR (#2983), p-mTOR (#4060), p-p70S6 (#9234), cleaved-caspase12(#2202), HDAC1-7 (#5356, #5113, #3949, #3443, #7558, #33418), Acetyl-Histone H3 (#4243), Histone H3 (#4499), CyclinD1 (#55506), Zo-1 (#13663), E-cadherin (#3195), β-Catenin (#8480), Slug(#9585), Snail(#3879), Beclin1(#3495), Atg5(#9980), Atg7(#8558), Atg12 (#8558), P62 (#8025), JNK (#9252), p-JNK (#4668), p-4EBP (#13443), p-GSK-3β (#5558), Erk (#4695), p-Erk (#4370), IRE1α (#3294), IKKα(#11930S), Phospho-IKKα/β(#2697S), Phospho-NF-κB p65(#3031S), NF-κB p65 (#4764S), Phospho-IκBα (#2859S), IκBα (#4812S). The following antibodies were purchased from Abcam: cleaved-caspase3 (ab214430), cleaved-PARP (ab32064), γ-H2AX (ab81299), CyclinB1 (ab181593), lipocalin-2 (LCN2, ab125075), ULK1 (ab177472), p-ULK1 (ab229909). LC3B (L7543) was purchased from Sigma-Aldrich and p-IRE1α (NB100-2323) from Novus. GAPDH and Tubulin were purchased from Proteintech.

**Supplementary Material 2:**

**Method:**

**Transfection**

Double-stranded siRNAs of ULK1, IRE1α, and LCN2 (GenePharma, Shanghai, China) were transfected into ESCC cells (KYSE-450 and KYSE-510) in a 6-well plate for 48 h using Lipofectamine 3000 (Invitrogen, Carlsbad, CA, USA) based on the manufacturer’s instructions. The knockdown efficacy was determined by western blot.

LCN2 was overexpressed in ESCC cells (KYSE-450 and KYSE-510) through lentivirus transduction. For lentiviral production, 85–90% confluent HEK-293T cells were transfected with a plasmid in a 10-cm dish, following the manufacturer’s protocols. Specifically, the primers of the targeted plasmid, PLVX-IRES-PURO-LCN2, are shown in **Table 3**. The plasmid packaging system included Δ8.9, VSVG, and PEI. After changing the RPMI 1640 with Opt-MEM (Gibco, NY, USA), all these elements were mixed and added into the medium for 4 h. Then the cell medium was replaced with RPMI 1640. The supernatant was collected 48 h post-transfection; debris was removed by centrifugation at 3,000 rpm for 10 minutes and filtered through a 0.45-μm filter before use. The supernatant containing lentiviral particles was used to transduce ESCC cells (KYSE-450 and KYSE-510) with 8 μg/mL polybrene. Selection of cells stably overexpressing LCN2 was performed using 2.5 μg/mL puromycin after 48 h infection for at least two weeks. The overexpression efficacy of LCN2 was determined by western blot.

**Table 3 Primers used for knocking down or over-expressing in ESCC cells**

| Gene | Primer Sequence（5′-3′） |
| --- | --- |
| si-ULK1 | Sense-1: CCAGGAAAUGGCUAAUUCUTT  Antisense-1: AGAAUUAGCCAUUUCCUGGTT  Sense-2: GGUACCUCCAGAGCAACAUTT  Antisense-2: AUGUUGCUCUGGAGGUACCTT |
| si-LCN2 | Sense-1: CCCACCUUGUCUGCUAAAUTT  Antisense-1: AUUUAGCAGACAAGGUGGGTT  Sense-2: GAGCUGACUUCGGAACUAATT  Antisense-2: UUAGUUCCGAAGUCAGCUCTT |
| si-IRE1 | Sense-1: GGUGCCACGAGUGAUUUAATT  Antisense-1: UUAAAUCACUCGUGGCACCTT  Sense-2：CAGCCAUCUACUGGAUAAATT  Antisense-2: UUUAUCCAGUAGAUGGCUGTT |
| OE-LCN2 | Sense: CGGAATTCATGCCCCTAGGTCTCCTGTGGC  Antisense: CGGGATCCTCAGCCGTCGATACACTGGTCG |
